# ATM Promotes the Obligate XY Crossover and both Crossover Control and Chromosome Axis Integrity on Autosomes

**DOI:** 10.1371/journal.pgen.1000076

**Published:** 2008-05-23

**Authors:** Marco Barchi, Ignasi Roig, Monica Di Giacomo, Dirk G. de Rooij, Scott Keeney, Maria Jasin

**Affiliations:** 1 Developmental Biology Program, Memorial Sloan-Kettering Cancer Center, New York, New York, United States of America; 2 Molecular Biology Program, Memorial Sloan-Kettering Cancer Center, New York, New York, United States of America; 3 Department of Endocrinology and Metabolism, Faculty of Science, Utrecht University, The Netherlands; 4 Center for Reproductive Medicine, Academic Medical Center, Amsterdam, The Netherlands; 5 Weill Graduate School of Medical Sciences of Cornell University, New York, New York, United States of America; Stowers Institute for Medical Research, United States of America

## Abstract

During meiosis in most sexually reproducing organisms, recombination forms crossovers between homologous maternal and paternal chromosomes and thereby promotes proper chromosome segregation at the first meiotic division. The number and distribution of crossovers are tightly controlled, but the factors that contribute to this control are poorly understood in most organisms, including mammals. Here we provide evidence that the ATM kinase or protein is essential for proper crossover formation in mouse spermatocytes. ATM deficiency causes multiple phenotypes in humans and mice, including gonadal atrophy. Mouse *Atm^−/−^* spermatocytes undergo apoptosis at mid-prophase of meiosis I, but *Atm^−/−^* meiotic phenotypes are partially rescued by *Spo11* heterozygosity, such that ATM-deficient spermatocytes progress to meiotic metaphase I. Strikingly, *Spo11^+/−^Atm^−/−^* spermatocytes are defective in forming the obligate crossover on the sex chromosomes, even though the XY pair is usually incorporated in a sex body and is transcriptionally inactivated as in normal spermatocytes. The XY crossover defect correlates with the appearance of lagging chromosomes at metaphase I, which may trigger the extensive metaphase apoptosis that is observed in these cells. In addition, control of the number and distribution of crossovers on autosomes appears to be defective in the absence of ATM because there is an increase in the total number of MLH1 foci, which mark the sites of eventual crossover formation, and because interference between MLH1 foci is perturbed. The axes of autosomes exhibit structural defects that correlate with the positions of ongoing recombination. Together, these findings indicate that ATM plays a role in both crossover control and chromosome axis integrity and further suggests that ATM is important for coordinating these features of meiotic chromosome dynamics.

## Author Summary

Meiosis is the specialized cell division that gives rise to reproductive cells such as sperm and eggs. During meiosis in most organisms, genetic information is exchanged between homologous maternal and paternal chromosomes through the process of homologous recombination. This recombination forms connections between homologous chromosomes that allow them to segregate accurately when the meiotic cell divides. Recombination defects can result in reproductive cells with abnormal chromosome numbers, which are a major cause of developmental disorders and spontaneous abortions in humans. Meiotic recombination is tightly controlled such that each pair of chromosomes undergoes at least one crossover recombination event despite a low average number of crossovers per chromosome. Moreover, multiple crossovers on the same chromosome tend to be evenly and widely spaced. Mechanisms of this control are not well understood, but here we provide evidence that ATM protein is required for normal operation of this process(es) in male mice. ATM has long been known to be involved in cellular responses to DNA damage. Our studies reveal a new function for this protein and also provide new insight into the mechanisms by which meiotic cells ensure accurate transmission of genetic material from one generation to the next.

## Introduction

Crossing-over between homologous chromosomes in conjunction with sister chromatid cohesion provides physical connections necessary for accurate chromosome segregation during the first meiotic division [1]. Due to their central role in meiosis, crossovers are tightly controlled in most organisms such that each chromosome pair gets at least one crossover, and multiple crossovers on the same chromosome tend to be evenly and widely spaced [2],[3]. One example of this control is the fact that non-exchange chromosomes are very rare even though the average number of crossovers per chromosome pair is low (often only 1–2 per pair). This observed tendency for at least one crossover to form per pair of homologous chromosomes is often referred to as the “obligate” crossover [3]. (The obligate crossover is viewed as one of the outcomes of the process(es) through which most crossovers form, not as a special type of crossover.) An especially striking example of this phenomenon is the sex chromosomes in males of many mammalian species, for which recombination between the X and Y is restricted to a relatively short region of homology, the pseudoautosomal region or PAR, which is ∼700 kb in some mouse strains [4]. Because a crossover must be formed to ensure segregation of the X and Y, the crossover rate per Mb of DNA is orders of magnitude higher in the PAR than in other regions of the genome.

A second manifestation of the regulation of crossing-over is interference, in which crossing-over in one genomic region makes it less likely that another crossover will be found nearby [2],[3],[5],[6]. A third manifestation is crossover homeostasis, documented in budding yeast as a tendency for crossover numbers to be maintained despite reduction in the number of recombination initiation events [7].

The number and distribution of crossovers are thus subject to multiple layers of regulation, which include both crossover-promoting (e.g., the obligate crossover and crossover homeostasis) and crossover-suppressing (e.g., interference) aspects. The term “crossover control” is often used as a catchall phrase to encompass these distinct aspects [3]. The various manifestations of crossover control may reflect a single underlying mechanism or closely interrelated set of mechanisms, although this remains to be experimentally verified ([2],[3],[5],[8] but see also [9]). The biochemical and genetic factors that govern crossover number and distribution are not well understood in most organisms, including in mammals.

Although key for chromosome segregation, crossing-over between homologous chromosomes is just one outcome of meiotic recombination, since noncrossovers also occur. Meiotic recombination initiates with DNA double-strand breaks (DSBs) introduced by the SPO11 transesterase [10]. DSBs are nucleolytically processed and the strand exchange proteins RAD51 and its meiotic homolog DMC1 act on the resulting single-stranded DNA ends to promote strand invasion into intact homologous DNA. Evidence from *Saccharomyces cerevisiae*, for which the mechanisms of meiotic recombination are best understood, suggests that the crossover versus noncrossover decision is made at or about this step during meiotic prophase [11]. In mouse spermatocytes, noncrossovers are estimated to outnumber crossovers approximately 10 to 1, as inferred from the ratio of RAD51 foci to MLH1 foci, which apparently mark sites of crossing-over [12],[13].

In male mice, several different molecular defects cause apoptosis of spermatocytes at the same point in meiotic prophase, equivalent to mid-pachynema in normal males [14],[15]. These defects include failure to initiate meiotic recombination (*Spo11^−/−^*) [16],[17] and failure to repair SPO11-generated DSBs (*Dmc1^−/−^*) [18],[19]. Despite the similar timing of apoptosis, spermatocytes from these mutants appear to arrest at different stages of meiotic progression, such that *Spo11^−/−^* spermatocytes express markers of early to mid-pachynema, whereas *Dmc1^−/−^* spermatocytes primarily express earlier markers (mid to late zygonema) [20]. Epistasis analysis with *Spo11^−/−^* revealed that the apparently earlier arrest in *Dmc1^−/−^* spermatocytes is a response to unrepaired DSBs [20]. Although the timing of apoptosis is quite different in females, oocytes also display distinct DNA damage- dependent and independent responses, such that *Spo11^−/−^* oocytes progress further than *Dmc1^−/−^* oocytes [21].

Loss of the serine/threonine kinase ATM also causes defects in meiotic progression during prophase I [22]–[24]. ATM activates cell cycle checkpoints in response to DSBs in somatic cells [25], and orthologs of ATM and the related kinase ATR also serve checkpoint monitoring functions for defects in meiotic interhomolog recombination in several organisms, including budding yeast and *Drosophila* (reviewed in [26]). However, phenotypes of *Atm^−/−^* spermatocytes and oocytes in mice are similar in many ways to those of *Dmc1^−/−^* meiocytes, and epistasis analysis with *Spo11* mutation further reinforces this similarity [20],[21]. These findings strongly indicate that the loss of ATM impairs the repair of meiotic DSBs, suggesting that ATM plays a role in promoting meiotic recombination rather than only serving a monitoring function. This interpretation is consistent with other studies that demonstrate that ATM and/or ATR orthologs promote normal recombination patterns in unperturbed yeast and *Drosophila* meiosis [26]–[29], and also promote repair of DNA damage [25],[30] as well as basic chromosomal events [31] in non-meiotic mammalian and yeast cells. Precisely what meiotic processes are influenced by ATM in mammalian cells has been difficult to uncover, however, in part because progression through meiotic prophase I fails so catastrophically in *Atm^−/−^* mutants.

During our investigation of the epistatic relationship between *Spo11* and *Atm*, we found that the testis cellularity of ATM-deficient mice was markedly increased by *Spo11* heterozygosity, accompanied by significantly improved chromosome synapsis. A similar finding with a different *Spo11* mutation was recently reported [32]. *Spo11^+/−^Atm^−/−^* spermatocytes can progress to meiotic metaphase I, although most cells undergo apoptosis at this stage. The rescue of meiotic progression to this stage allowed us to further explore the role of ATM in meiosis. Our analysis provides evidence for involvement of ATM in several aspects of crossover control and chromosome axis integrity.

## Results

### Spermatocyte Apoptosis at Metaphase I in *Spo11^+/−^Atm^−/−^* Mice

**Figure 1:**
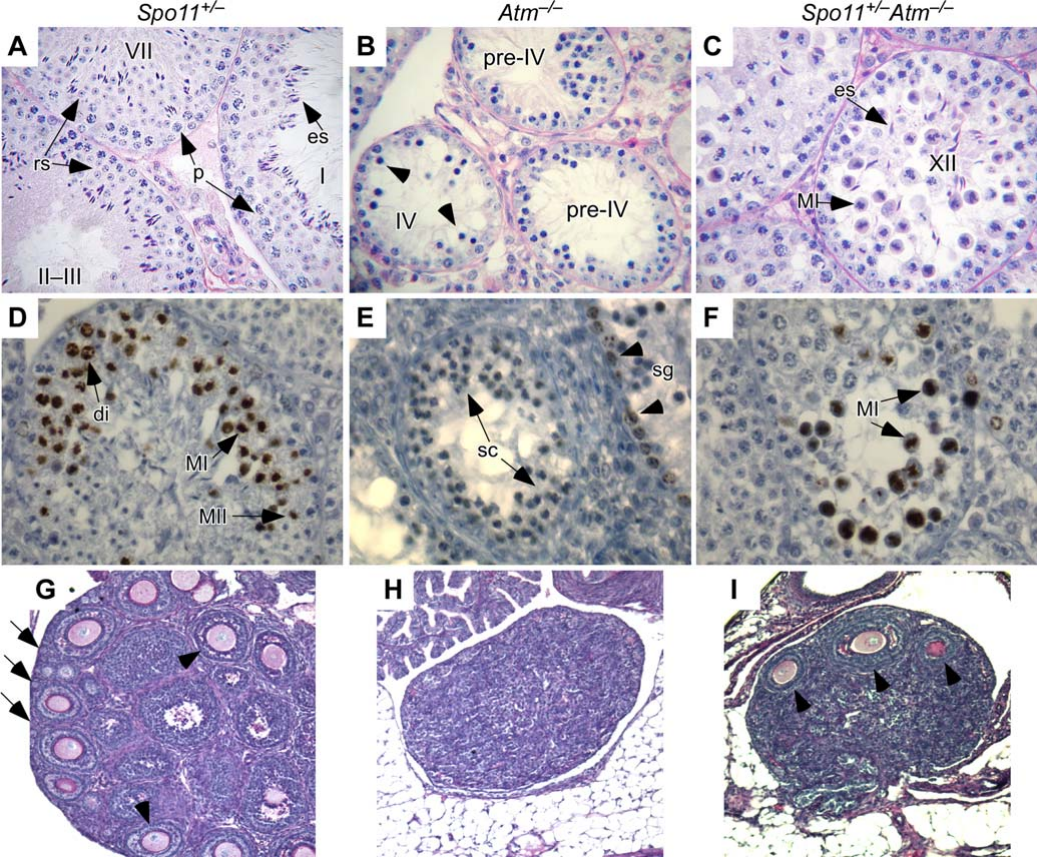
*Spo11* heterozygosity partially ameliorates defects in meiotic progression in the absence of ATM. (A–C) Periodic acid Schiff (PAS)-stained testis sections from mice of the indicated genotypes. Roman numerals denote stages of the seminiferous epithelial cycle [33]. *Spo11^+/−^* mice (A) show normal patterns, with examples of pachytene spermatocytes (p), round spermatids (rs), and elongating spermatids (es) indicated. *Atm^−/−^* seminiferous tubules (B) show decreased cellularity as a result of spermatocyte apoptosis at stage IV. Cells that appear to be apoptotic are indicated (arrowheads). *Spo11^+/−^Atm^−/−^* tubules (C) have increased testis cellularity compared to *Atm^−/−^* mice. Examples are indicated of metaphase I spermatocytes (MI) and elongating spermatids (es) in stage XII tubules. The cytosol of many metaphase cells is intensely stained, suggesting that these cells are undergoing apoptosis (see also Figure S1). (D–F) Anti-phospho-histone H3 (p-H3) staining of testis sections. Presence of p-H3 is marked by a dark brown enzymatic precipitate. Examples of spermatogonia (sg) and spermatocytes in diplonema (di), metaphase I (MI), or metaphase II (MII) are indicated. Examples of the most advanced non-apoptotic spermatocytes observed in *Atm^−/−^* are indictated in panel E (sc). (G–I) Reduced *Spo11* gene dosage partially rescues oogenesis in *Atm^−/−^* mice. Ovary sections from 17–18 dpp mice of the indicated genotypes were stained with PAS. In *Spo11^+/−^* mice (G), as in wild type, growing and antral follicles (arrowheads) and primordial follicles at the cortex of the ovary (arrows) are observed. Essentially no follicles at any stage of maturation are present in *Atm^−/−^* ovaries (H), whereas some growing and antral follicles (arrowheads) are observed in *Spo11^+/−^Atm^−/−^* ovaries (I).

Testis cellularity of ATM-deficient mice is markedly increased by *Spo11* heterozygosity [32]. To characterize the increase, we performed a histological analysis of testis sections. Seminiferous tubules contain germ cells at various stages of spermatogenesis, with mitotic and early meiotic cells at the base of the tubule and later meiotic and post-meiotic stages displaced toward the lumen. Tubule cross sections can be classified into stages, referred to as I–XII, based on the particular set of germ cells present [33]. *Spo11^+/−^* testes show the normal pattern of these various stages (Figure 1A and data not shown), whereas tubules in *Atm^−/−^* mice are severely depleted of cells as a result of apoptosis of pachytene spermatocytes at stage IV [20],[23] (Figure 1B). In contrast, *Spo11^+/−^Atm^−/−^* mice presented morphologically normal pachytene cells in tubules at stage IV and beyond (Figure 1C and Figures S1A and S1B). Although some apoptosis at stage IV was still observed (data not shown), most *Spo11^+/−^Atm^−/−^* spermatocytes appeared to reach metaphase (stage XII tubules, Figure 1C). Round and elongating spermatids and sperm were also observed, although post-meiotic stages were severely reduced in number compared to wild-type mice, and in some cases appeared abnormal (Figures 1C and S1B; data not shown). Meiotic progression is dependent on *Spo11* heterozygosity, as *Spo11^−/−^Atm^−/−^* mice undergo a stage IV apoptosis, like *Atm^−/−^* mice [20].

To further evaluate meiotic progression, testis sections were stained for phospho-histone H3 (p-H3), which is normally detected in spermatocytes from diplonema through the second division, as well as in dividing spermatogonia [34] (Figure 1D). *Atm^−/−^* spermatogonia were positive for p-H3 but spermatocytes were not, as expected because of apoptosis during prophase I (Figure 1E and data not shown). By contrast, p-H3-positive spermatocytes were observed in *Spo11^+/−^Atm^−/−^* mice, verifying progression to metaphase I (Figure 1F). These metaphase I cells of *Spo11^+/−^Atm^−/−^* mice often showed relatively darkly stained cytoplasm characteristic of apoptosis (Figure 1F and data not shown). TUNEL staining confirmed that most spermatocytes were eliminated at metaphase I by apoptosis [32] (Figure S1C). Thus, *Spo11* heterozygosity sufficiently rescued defects associated with ATM loss to allow progression to metaphase I, but spermatogenesis was for the most part halted at this stage.

### Partial Amelioration of *Atm^−/−^* Meiotic Phenotypes by *Spo11* Heterozygosity in Females

Loss of ATM also leads to germ cell depletion in females [22],[24]. We examined ovaries of *Spo11^+/−^Atm^−/−^* mice to determine if meiotic progression could also be rescued in oocytes. Ovaries were examined between 17 and 29 dpp, at a time when wild-type or *Spo11^+/−^* ovaries contain several thousand oocytes (Figure 1G). In *Atm^−/−^* females, only one oocyte was found in four mice examined (0.13 oocytes/ovary) (Figure 1H), whereas in four *Spo11^+/−^Atm^−/−^* females, there was a small but significant increase to 7.9±3.7 follicular oocytes/ovary (Figure 1I; mean±sd, *p*<0.0001, *t* test). Thus, *Spo11* heterozygosity partially suppresses *Atm^−/−^* meiotic defects in both males and females, although to a different extent in females.

### Defects in Forming the Obligate Crossover on Sex Chromosomes in the Absence of ATM

**Figure 2:**
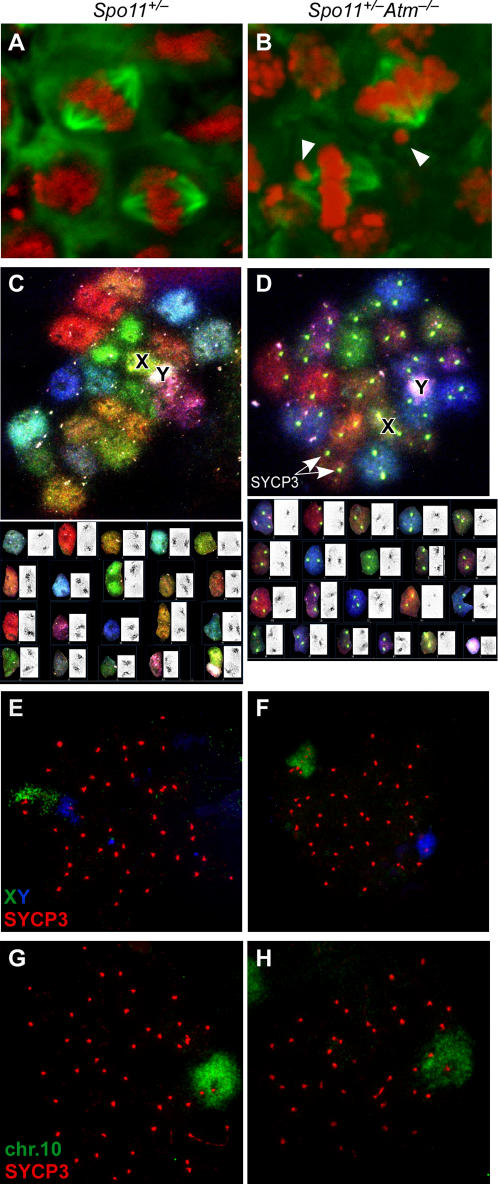
ATM-deficient spermatocytes show metaphase I lagging chromosomes and defects in forming the obligate crossover between the sex chromosomes. (A, B) Lagging chromosomes in *Spo11^+/−^Atm^−/−^* spermatocytes. Testis sections were immunostained with anti-tubulin antibodies to detect the spindle (green) and with DAPI (pseudocolored red). Arrowheads show examples of lagging chromosomes. (C, D) SKY of metaphase I chromosomes showing univalent X and Y in *Spo11^+/−^Atm^−/−^* (D) but not *Spo11^+/−^* (C) spermatocytes. Lower panels show karyotypes of the metaphases, with the inverted DAPI chromosome images in insets. Centromeres are visible as dark masses in the insets and as bright spots in (D), where anti-SYCP3 staining was included. (E–H) Metaphase I chromosome spreads from *Spo11^+/−^* (E, G) or *Spo11^+/−^Atm^−/−^* (F, H) were analyzed by FISH for the indicated chromosomes in conjunction with immunofluorescence for SYCP3. At metaphase I, SYCP3 is retained almost exclusively at the centromeric regions (although note that SYCP3 is also present as discrete foci along the X chromosome).

To further characterize the metaphase I defect of *Spo11^+/−^Atm^−/−^* spermatocytes, we examined meiotic spindles in testis sections (Figure 2A and 2B). Well-developed bipolar spindles were apparent in both *Spo11^+/−^* and *Spo11^+/−^Atm^−/−^* mice, with chromosome congression at the metaphase plate. However, one or two lagging chromosomes were often evident in the *Spo11^+/−^Atm^−/−^* mice (arrowheads, Figure 2B). Specifically, two of 11 *Spo11^+/−^* spindles (18%) showed a single lagging chromosome, whereas 11 of 13 *Spo11^+/−^Atm^−/−^* spindles (85%) showed lagging chromosomes (eight with one laggard, two with two laggards, one with three laggards) (*p* = 0.0031, Fisher's exact test). One possible explanation of these results is the frequent presence of achiasmate chromosomes (i.e., which have not undergone crossing over), because crossing-over is required for chromosome congression at metaphase I [35].

To determine if a particular chromosome pair was more likely to be achiasmate, we performed spectral karyotyping on meiotic chromosome spreads. In three *Spo11^+/−^* metaphases examined, each chromosome pair was present as a single unit (a bivalent) including the XY pair (Figure 2C). Autosomes also formed bivalents in five *Spo11^+/−^Atm^−/−^* metaphases examined, but the sex chromosomes were separated (formed univalents) in four of the cells (Figure 2D). To specifically examine the sex chromosomes in a larger number of metaphases, we performed FISH with probes for the X and Y. The sex chromosomes were always joined in *Spo11^+/−^* spermatocytes, as expected (*n* = 10; Figure 2E), but were univalents in 80% of *Spo11^+/−^Atm^−/−^* spermatocytes (Figure 2F) (*n* = 20; *p*<0.0001, Fisher's exact test). This behavior contrasted with autosomes: a single FISH signal was observed in both genotypes for Chromosome 10 (Chr10) (*n* = 15 for each genotype) (Figure 2G and 2H) and for Chr3 (data not shown). These results indicate that chiasma formation is not globally defective, but suggest instead that the XY pair is uniquely sensitive to defects caused by lack of ATM.

### Impaired Sex Chromosome Synapsis

**Figure 3:**
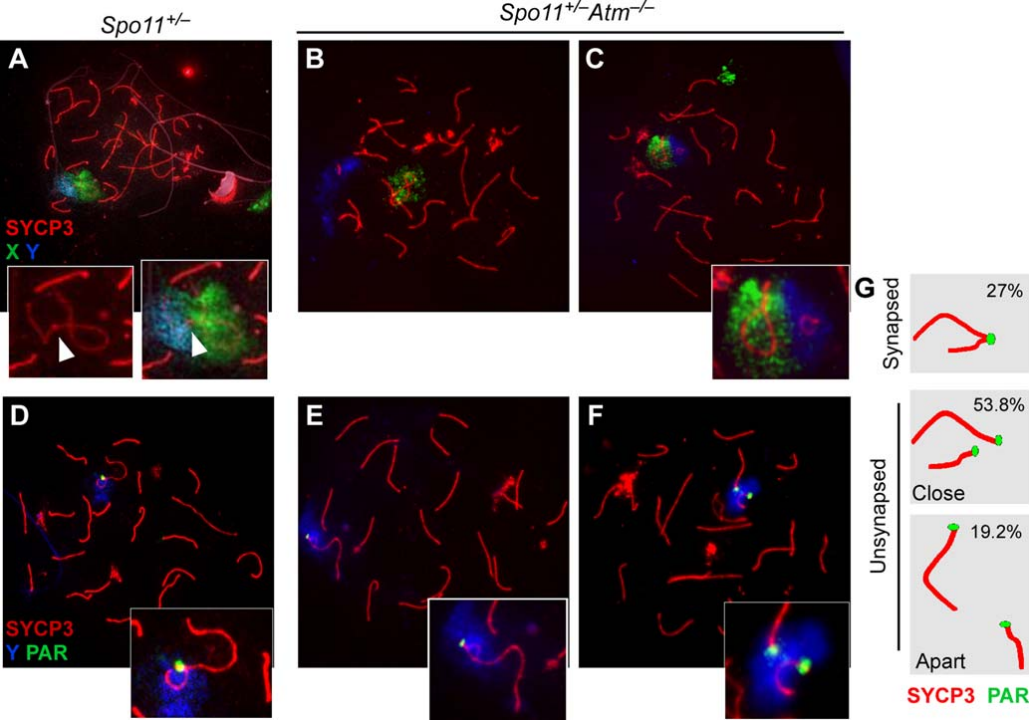
Aberrant sex chromosome synapsis in the absence of ATM. Pachytene chromosome spreads from *Spo11^+/−^* (A, D) and *Spo11^+/−^Atm^−/−^* (B, C, E, F) were analyzed by immunofluorescence for SYCP3 along with FISH for X and Y (A–C) or Y and the PAR (D–F). Insets show the disposition of the X and Y. Arrowheads in (A) point to the synapsed PAR. (G) Frequencies of various sex chromosome configurations in *Spo11^+/−^Atm^−/−^* spreads based on FISH for Y and PAR. XY pairs were scored as “synapsed” if they showed a single PAR signal and intimately associated axes (e.g., panels D, E). If two PAR signals were observed, the X and Y were scored as “unsynapsed” and were further classified as to whether the chromosomes were close to one another in the spread (e.g., panel F) or far apart (similar to panel B).

During meiotic prophase, homologous chromosomes are juxtaposed along their length via the synaptonemal complex (SC), which is fully assembled by pachynema. The SC comprises several proteins, including the axial element protein SYCP3, which assembles beginning in leptonema, and the central element protein SYCP1, which assembles along chromosome axes as homologous chromosomes synapse beginning in zygonema. Crossing-over is intimately associated with SC formation (reviewed in [36]). We therefore tested whether the XY crossover defect is accompanied by a defect in synapsis. As expected, the X and Y were always adjacent to each other in *Spo11^+/−^* pachytene spermatocytes (*n* = 38 cells) (Figure 3A), and the chromosome axes were closely juxtaposed at one end (Figure 3A insets), indicative of synapsis within the context of the SC. In contrast, the X and Y were far apart in 10.1% of *Spo11^+/−^Atm^−/−^* spermatocytes (*n* = 89 cells; Figure 3B). Furthermore, even though the X and Y were adjacent to one other in the remaining *Spo11^+/−^Atm^−/−^* cells, they were frequently not synapsed, as judged by the absence of intimate contact between their respective axes (Figure 3C inset). Separate immunostaining experiments with anti-SYCP1 and anti-SYCP3 confirmed that PAR synapsis occurred normally in *Spo11^+/−^* spermatocytes but was frequently defective in *Spo11^+/−^Atm^−/−^* cells (data not shown).

The sex chromosomes share homology only within the PAR, which is where the obligate XY crossover occurs (see Introduction). We used FISH to more precisely characterize PAR pairing and synapsis. In every *Spo11^+/−^* spermatocyte examined (*n* = 50 cells), a single, merged PAR signal that overlapped intimately juxtaposed axes was observed for the X and Y, consistent with synapsis in this region (Figure 3D). In contrast, three XY configurations were observed in *Spo11^+/−^Atm^−/−^* spermatocytes (Figure 3E and 3F; summary in Figure 3G). In 27% (*n* = 78 cells), there was a single PAR signal that overlapped intimately juxtaposed axes, consistent with XY synapsis (Figure 3E). However, in the major class (53.8%), PAR signals were separated even though the X and Y were adjacent (Figure 3F). Thus, even though the X and Y were usually juxtaposed, the PARs usually failed to synapse. The remaining 19.2% of spermatocytes had well separated X and Y (similar to Figure 3B; data not shown). These findings reveal that ATM is required for efficient pairing and/or synapsis of the sex chromosomes. As described further in Discussion, we consider it likely that the small size of the available region of homology within the PAR makes this genomic region uniquely sensitive to defects in these processes.

Importantly, the absence of ATM did not significantly reduce the total number of RAD51 foci in leptotene and zygotene spermatocytes in a *Spo11^+/−^* background. We observed 144.0±31.0 in *Spo11^+/−^* (14 cells) versus 123.5±78.1 in *Spo11^+/−^Atm^−/−^* in leptonema (25 cells) (mean±sd, *p* = 0.354, *t* test); and 173.8±23.8 in *Spo11^+/−^* (20 cells) versus 202.7±57.2 in *Spo11^+/−^Atm^−/−^* (23 cells) in zygonema (*p* = 0.041). This result suggests that the increased frequency of asynaptic and/or achiasmate sex chromosomes cannot be attributed simply to a reduction in overall DSB frequencies in *Spo11^+/−^Atm^−/−^* compared to *Spo11^+/−^*.

### Both ATM and XY Synapsis Are Dispensable for Meiotic Sex Chromosome Inactivation

**Figure 4:**
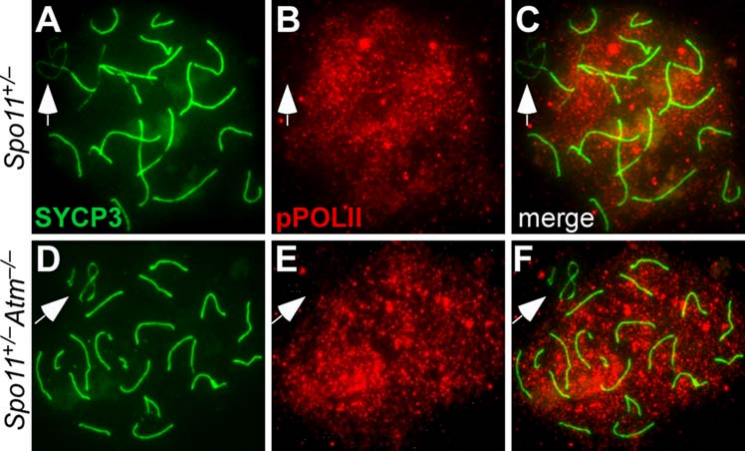
ATM and XY chromosome synapsis are dispensable for meiotic sex chromosome inactivation. Pachytene chromosome spreads were stained for SYCP3 and phosphorylated RNA polymerase II (pPOLII). In both *Spo11^+/−^* (A–C) and *Spo11^+/−^Atm^−/−^* (D–F), phosphorylated RNA polymerase II is excluded from the sex chromatin (arrows).

Sex chromosomes in spermatocytes are transcriptionally silenced during prophase through meiotic sex chromosome inactivation (MSCI), during which the X and Y are included in the sex body, a heterochromatin domain that excludes the active (phosphorylated) form of RNA polymerase II (reviewed in [37],[38]) (Figure 4A–4C). Phosphorylated RNA polymerase II was excluded from the sex chromatin of both *Spo11^+/−^* and *Spo11^+/−^Atm^−/−^* spermatocytes (Figure 4D–4F), indicating that ATM is dispensable for MSCI. Importantly, MSCI occurred even when the X and Y did not synapse within the PAR (Figure 4), consistent with recent studies suggesting that MSCI is driven at least in part by asynapsis per se [39].

The sex body is enriched for numerous proteins and protein posttranslational modifications [40]. One of these modifications is phosphorylation of the histone variant H2AX (γH2AX), which has also been implicated in MSCI [41]. ATM is dispensable for γH2AX formation in the sex body [32]. Using FISH, we confirmed that the sex chromosomes were included within a γH2AX-positive domain even when they were not synapsed (Figure S2A and S2B). When the sex chromosomes were separated, the X and Y were contained within separate γH2AX domains (Figure S2C and S2D). Similarly, neither ATM nor PAR synapsis were required for localization of two additional sex body components, NBS1 and TOPBP1 (Figure S2E and S2F). Thus, ATM is dispensable for formation of apparently bona fide sex bodies.

### Chromosome Structure Defects on Autosomes in *Spo11^+/−^Atm^−/−^* Spermatocytes

#### Chromosome Axis Gaps and Fragmentation

**Figure 5:**
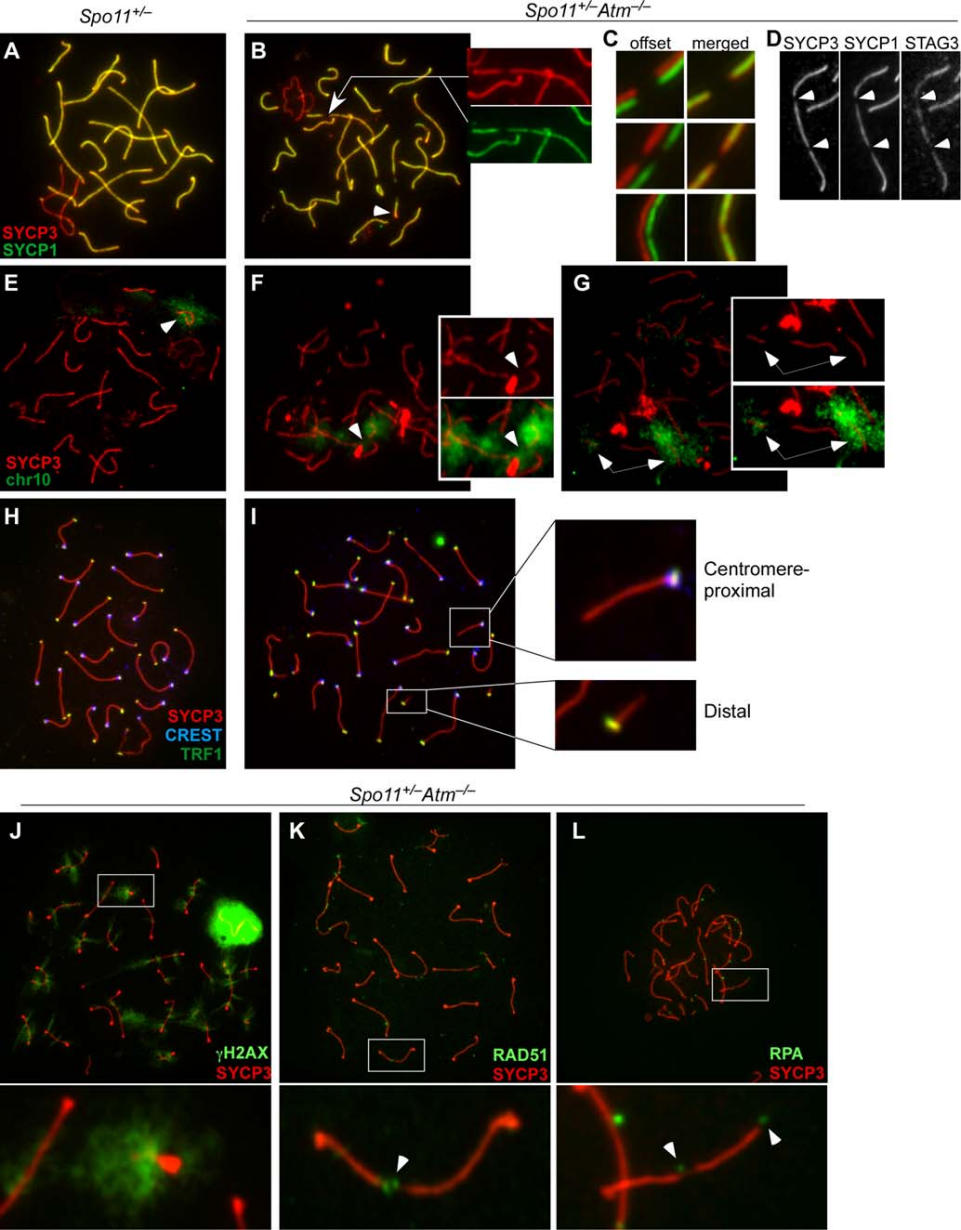
Chromosome structure defects in *Spo11^+/−^Atm^−/−^* spermatocytes. (A–C) Axial element (SYCP3) and central element (SYCP1) defects in pachytene spermatocytes from *Spo11^+/–^Atm^–/–^* mice. Chromosome spreads from *Spo11* ^+/–^ (A) and *Spo11^+/–^Atm^–/–^* spermatocytes (B–C) were immunostained with anti-SYCP3 and anti-SYCP1 antibodies. Insets in (B) highlight defects visible on some autosomes (arrow). Additional examples are shown in (C), which shows red and green immunofluorescence channels offset (left) or merged (right). (D) Colocalization of defects in SYCP3 and SYCP1 staining with gaps in staining for the meiotic cohesin subunit STAG3 in *Spo11^+/−^Atm^−/−^* spermatocytes. (E–G) Axial defects in *Spo11^+/−^Atm^−/−^* pachytene spermatocytes can be associated with chromosome fragmentation. Anti-SYCP3 immunofluorescence (red) and FISH for chr10 (green) was combined on spermatocyte spreads. In *Spo11* ^+/−^ (E), SYCP3 and chr10 signals are always continuous. In *Spo11^+/−^Atm^−/−^*, examples are shown of SYCP3 gaps that either are not (F) or are (G) associated with overt fragmentation of chr10. (H, I) Short SC fragments are the distal ends of chromosomes. Spread pachytene nuclei from *Spo11^+/−^* (H) and *Spo11^+/−^Atm^−/−^* (I) were stained for SYCP3 (red) and the telomeric protein TRF1 (green), along with CREST antibodies to detect centromeres (blue). Insets in (I) show a short SC fragment from a distal chromosome end showing telomeric staining at one end (lower inset) and a longer SC fragment from a centromere-proximal chromosome end showing colocalization of the telomere and centromere (upper inset). (J–L) Colocalization of chromosome axis defects with markers of sites of DSB repair. *Spo11^+/−^Atm^−/−^* spermatocyte spreads were stained with anti-SYCP3 (red) and with antibodies to either γH2AX (J; 43 cells analyzed from 2 mice), RAD51 (K; 51 cells from 3 mice), or RPA (L; 42 cells from 2 mice). Insets show enlargements of the boxed regions of the spreads. Arrowheads point to examples of RAD51 or RPA foci that colocalize with SC gaps.

*Atm^−/−^* mice, like several other meiotic mutants including *Spo11^−/−^*, have profound chromosome synapsis defects [22],[24], but *Spo11^+/−^Atm^−/−^* pachytene spermatocytes have well-synapsed chromosomes [32] (Figure 5). Nonetheless, numerous chromosome structure abnormalities were present. Thinning and gaps in the chromosome axes, as evidenced by SYCP3 staining, were frequently observed (68/475 or 14.3% of bivalents, 96 cells) (Figure 5B and 5C). When SYCP3 abnormalities were present, they were always associated with thinning or gaps in SYCP1 (Figure 5C), although SYCP3 staining was not always noticeably perturbed in cases where SYCP1 staining showed thinning or gaps (29/83 or 31% of chromosomes containing SYCP1 abnormalities) (Figure 5C, lowest panel). We also examined staining for STAG3, a meiosis-specific cohesin subunit that is axis-associated. Even in fully wild-type spermatocytes, anti-STAG3 antibodies show a staining pattern that is less continuous and more uneven than anti-SYCP3 staining (data not shown), but importantly, SYCP3 staining anomalies in *Spo11^+/−^Atm^−/−^* spermatocytes were nearly always associated with gaps in the STAG3 staining (56 of 61 SYCP3 anomalies, or 91.8%) (Figure 5D).

In addition, more than the normal 19 autosomal SCs were often apparent, due to the presence of supernumerary, short SC fragments (arrowhead, Figure 5B). In a representative experiment, 55 short SC fragments were observed in 33 pachytene spermatocytes. Chromosome fragmentation was previously observed in metaphase spreads of *Spo11^+/−^Atm^−/−^* spermatocytes [32]. The current findings reveal that structural abnormalities are already present in pachynema, if not earlier.

To further characterize the SC gaps and fragments, we examined individual chromosomes using combined FISH and immunofluorescence. In each of 20 *Spo11^+/−^* spermatocytes examined, Chr10 and SYCP3 signals were both uninterrupted, as expected (Figure 5E). Most *Spo11^+/−^Atm^−/−^* spermatocytes also showed uninterrupted SYCP3 and Chr10 FISH signals (data not shown), but a gap in SYCP3 staining was observed in 11 of 57 cells examined (19.3%). For these cells, about half still had a continuous Chr10 FISH signal even though there was a gap in the SYCP3 staining (10.5% of total) (Figure 5F). The overall continuity of these bivalents suggests that the DNA continuity is preserved (at least for a subset of the four chromatids) and that it is instead the continuity of axial and/or SC structure that is disrupted. Interestingly, each of the remaining spermatocytes had two separated chr10 fragments (8.8% of total) (Figure 5G). Analysis of chr3 gave similar results (data not shown). These findings suggest the possibility that chromosome axis defects (discontinuities in SYCP3 staining) were often associated with compromised chromosome integrity leading to overt chromosome fragmentation. A possible explanation for these observations is that “weak spots” on axes are susceptible to disruptive forces from chromosome spreading procedures resulting in extreme stretching or even complete severing of the DNA of all four chromatids. Alternatively, stretching and/or chromatid severing occur in vivo, perhaps as a consequence of large-scale chromosome movements that have been documented during meiotic prophase in some organisms (e.g., [42].

Mouse chromosomes are acrocentric, that is, with the centromere near one end. To determine what part(s) of chromosomes made up the short SC fragments, spread nuclei were immunostained for the telomeric protein TRF1 [43] (Figure 5H and 5I) (56 cells). Most of the short SC fragments did not contain centromeres as judged by staining with CREST antibodies or with DAPI, but nearly all (94.2%, *n* = 155 fragments) showed TRF1 staining at one end (Figure 5I, lower inset). TRF1 detection efficiency on telomeres of full-length chromosomes was comparable (99.5%, *n* = 684 chromosomes). The spreads also contained longer SC fragments with just one telomere, and for these, the telomeric end also contained a centromere (Figure 5I, upper inset). Overall, centromere-containing SC fragments were significantly longer (5.2±2.3 μm) than non-centromeric SC fragments (2.7±2.0 μm, *p* = 0.0001, *t* test). We conclude that the short SC fragments were usually derived from the distal tips of chromosomes, i.e., that overt chromosome fragmentation occurs preferentially in distal regions.

#### Colocalization of Axis Defects with Sites of Double-Strand Break Repair

One role of ATM in early meiotic prophase is to promote the phosphorylation of H2AX in response to SPO11-generated DSBs [20],[32]. In *Atm^−/−^* spermatocytes, γH2AX is nearly absent in leptonema and much reduced in zygonema [20],[32], and similar patterns have been shown in *Spo11* ^+/−^ *Atm^−/−^* spermatocytes [32] (see Figure S3A, S3B, S3C, and S3D), indicating that rescue of meiotic progression is not associated with restoration of normal patterns of H2AX phosphorylation. It is important to note, however, that even in the absence of ATM, some γH2AX does form on autosomes in response to SPO11-generated DSBs, presumably due to the activity of the ATM-related kinase ATR [20],[32].

As previously noted [32], pachytene and diplotene *Spo11^+/−^Atm^−/−^* spermatocytes showed bright puffs of γH2AX staining at both interstitial and telomeric positions on chromosome axes that had normal SYCP3 patterns (80/570 chromosomes  = 14.0%; 43 cells) (Figures 5J and S3F). Such staining is aberrant because γH2AX largely disappears from the autosomes by mid-pachynema in normal spermatocytes [44] (Figure S3E). These results, along with persistent foci of DSB repair proteins such as RAD51 [32] (Figure 5K) and the single-strand binding protein RPA (Figure 5L), suggest the presence of persistent DSBs (or de novo DSB formation) in *Spo11* ^+/−^ *Atm^−/−^* spermatocytes. Importantly, many SYCP3 abnormalities were associated with these cytological markers of DSB repair: γH2AX was present at 59% of SYCP3 gaps (*n* = 66 gaps) and at the non-telomeric ends of 72.3% of short SC fragments (*n* = 101 fragments) (Figure 5J); RAD51 foci were present at 38.2% of SYCP3 gaps (*n* = 68) (Figure 5K) and at the non-telomeric ends of 50% of short SC fragments (*n* = 62); and RPA foci were present at 50.9% of gaps (*n* = 114) and at the non-telomeric ends of 57.1% of short SC fragments (*n* = 91) (Figure 5L). The frequent association of SYCP3 anomalies with DSB markers suggests a possible mechanistic link between chromosome axis defects and the ongoing process of DSB repair (see Discussion).

#### Longer Autosomal Synaptonemal Complexes

On average, *Spo11^+/−^Atm^−/−^* spermatocytes showed a small (∼10%) increase in the total length of autosomal SCs compared to *Spo11^+/−^* littermates (respectively, 184.8±18.9 μm (38 cells) versus 167.7±16.3 μm (46 cells), *p* = 0.0002, *t* test). These figures are for total lengths of autosomal SYCP3 staining and thus are separate from effects of gaps or fragmentation. Indistinguishable results were seen in separate experiments where spreads were immunostained with anti-SYCP1 and anti-SYCP3 (data not shown). The increase in average length was seen for all sizes of autosome, from largest to smallest (Table S1). Note, however, that the size ranges overlapped, such that 29/38 (76.3%) of ATM-defective spermatocytes had total SC lengths within the range found in most *Spo11^+/−^* cells (140–200 μm) (Figure S4).

### Evidence for Defective Crossover Control on Autosomes in the Absence of ATM

**Figure 6:**
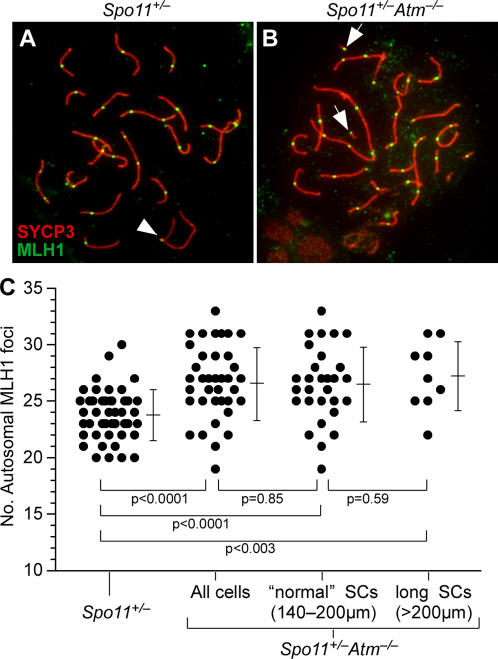
Elevated crossover numbers on autosomes in the absence of ATM. (A, B) MLH1 foci on pachytene chromosomes. Spermatocyte spreads of the indicated genotype were stained for MLH1 (green) and SYCP3 (red). Arrowhead in (A) points to an MLH1 focus visible on the PAR; arrows in (B) show examples of MLH1 foci on short SC fragments. The XY pair at 6:00 o'clock in the spread in (B) does not show an MLH1 focus. (C) Autosomal MLH1 focus counts (bars = mean±sd). For *Spo11^+/−^Atm^−/−^*, data are shown for all of the cells analyzed (total cells, *n* = 38), the subset of cells (*n* = 29) that had a total SC length within the range observed in >90% of *Spo11^+/−^* cells (140–200 μm), and the subset of cells with longer SCs (>200 μm). *P-*values are shown for Mann-Whitney U tests of the indicated pairwise comparisons. The average total SC length (174.4±12.4 μm) in the subset of *Spo11^+/−^Atm^−/−^* cells with “normal” length SCs was slightly greater (3.5%) than the average SC length observed in *Spo11^+/−^* (167.7±16.3 μm), but this difference was not statistically significant (*p* = 0.065, *t* test).

As described above, the XY pair frequently failed to generate a crossover in the absence of ATM, whereas crossing over on autosomes appeared grossly normal, at least insofar as ensuring formation of bivalents. This pattern could indicate that ATM is required specifically for recombination on the sex chromosomes, but the numerous structural defects on autosomes demonstrate that consequences of ATM loss are not confined to the sex chromosomes. We therefore considered the possibility that ATM deficiency alters crossing over more generally. To test this idea, we examined autosomal MLH1 foci, which localize to crossover-designated sites at pachynema [12],[13],[45] (Figure 6A). Autosomal MLH1 foci in *Spo11^+/−^Atm^−/−^*spermatocytes appeared grossly normal in that nearly all bivalents had at least one focus (Figure 6B), consistent with the metaphase I analysis indicating that ATM is not required for crossover formation per se. However, a close examination revealed several unusual characteristics consistent with a small but significant defect in crossover control on autosomes. These findings are in general accord with the recent demonstration of crossover control defects associated with mutations of *Mre11* and *Nbs1* that attenuate ATM signaling in mouse [46].

#### Elevation of Total Crossover Numbers

The total number of crossovers tends to vary relatively little between spermatocytes in an individual (e.g., [12],[47],[48]). This feature is likely a consequence of crossover control (especially interference) acting to limit the number of crossovers on a per-chromosome basis [3],[49]. We find that absence of ATM in a *Spo11^+/−^* background results in a significant increase in the number of autosomal MLH1 foci, suggesting in turn that crossover numbers are elevated: *Spo11^+/−^* spermatocytes had 23.8±2.2 foci (46 cells from 4 mice), whereas *Spo11^+/−^Atm^−/−^* spermatocytes had 26.7±3.2 foci (38 cells from 4 mice) (Figure 6C). This increase was also apparent in the range of the number of foci per cell, since up to 33 autosomal MLH1 foci were observed in *Spo11^+/−^Atm^−/−^* spermatocytes compared with a maximum of 30 in *Spo11^+/−^* mice (Figure 6C). The increased total number was due to an increase in the frequency of intact or gapped autosomes with >2 foci as well as the presence of short SC fragments with an MLH1 focus (*p* = 0.0006, G-test). No significant difference was observed in the number of autosomes without foci (*p* = 0.101, G test), although short SC fragments often lacked foci (59 of 143 fragments, 41.3%).

Previous studies have shown a correlation between SC length and crossover frequency (e.g., [47]). The reason for this correlation is not known, and in particular it is not clear that the correlation reflects a causal relationship between these two properties (see [50] for detailed discussion). Nevertheless, we considered whether the increased average SC length in *Spo11^+/−^Atm^−/−^* spermatocytes could account for increased numbers of MLH1 foci. As noted above, most ATM-deficient spermatocytes had SC lengths within the same size range observed in most *Spo11^+/−^* cells (140–200 μm total length). When this subset of *Spo11^+/−^Atm^−/−^* cells was considered separately, they showed the same increase in total MLH1 foci relative to *Spo11^+/−^* (26.5±3.3 foci per cell), indistinguishable from considering either the complete population of *Spo11^+/−^Atm^−/−^* cells analyzed (26.7±3.2) or the subset with SCs longer than 200 μm (27.2±3.0) (Figure 6C). We conclude that the increase in total MLH1 numbers in *Spo11^+/−^Atm^−/−^* spermatocytes is not simply a secondary consequence of the increased average SC length.

Different mouse chromosomes show different patterns for number and position of crossovers, with similarly sized chromosomes showing similar behavior [12],[51]. Because of these differences, small alterations in crossover patterns may be obscured when data are pooled for all chromosomes. To overcome this issue, we rank-ordered the autosomes in each spermatocyte spread based on SC length, then grouped similarly sized chromosomes together to form five groups (the two longest chromosomes together, the next three longest together, etc.; see Materials and Methods for more detail). For each size group, we then compared MLH1 numbers in *Spo11^+/−^Atm^−/−^* vs. *Spo11^+/−^* (Table 1). The increase in MLH1 numbers was most striking for chromosomes in the middle of the size distribution, namely, size ranks 6–11 and 12–16. Both of these groups showed a highly significant increase in the number of chromosomes with two or more foci (Table 1). Other chromosome groups also showed increases, but the differences did not reach the level of statistical significance. For example, the smallest three chromosomes only rarely form more than one crossover in normal spermatocytes [12],[51]. Consistent with the overall increase in MLH1 foci, examples of short chromosomes with two foci were observed more often in *Spo11^+/−^Atm^−/−^*, although this increase was not statistically significant (Table 1). These results suggest that absence of ATM increases crossover numbers over much of the genome, although some chromosomes seem to be more significantly affected than others.

**Table 1:** Numbers of MLH1 foci per chromosome.

Genotype	Chromosome Size Ranks	Bivalents with Two or More MLH1 Foci	MLH1 Foci per Bivalent				p
			3	2	1	0	
*Spo11^+/−^*	1–2	57.8%	1	51	37	1	
	3–5	57.8%	0	78	57	0	
	6–11	29.7%	1	79	182	7	
	12–16	8.9%	0	20	199	6	
	17–19	3.0%	0	4	124	4	
*Spo11^+/−^Atm^−/−^*	1–2	64.3%	4	32	20	0	0.14
	3–5	59.5%	1	49	32	2	0.35
	6–11	51.2%	2	84	80	2	6×10^−5^
	12–16	22.1%	0	31	106	3	5×10^−4^
	17–19	7.1%	0	6	74	4	0.16

Pachytene spermatocyte spreads were immunostained for SYCP3 and MLH1. Autosomal bivalents in each spread were rank-ordered according to SC length from 1 (largest) to 19 (smallest), then divided into groups of similarly sized chromosomes. *P*-values are shown for log-likelihood (G) tests comparing each size group from *Spo11^+/−^Atm^−/−^* cells to the same size group from *Spo11^+/−^* cells, not including the zero-focus class. (Note that bivalents with zero MLH1 foci are ambiguous because foci are transient.)

#### Decreased Cytological Interference

**Figure 7:**
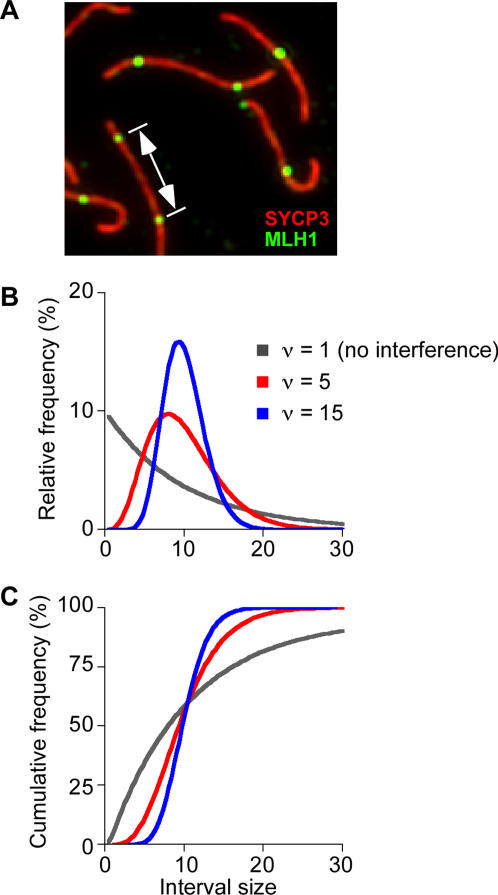
Measuring cytological interference between MLH1 foci. (A) Pachytene spermatocyte spreads were immunostained for SYCP3 (shown in red) and MLH1 (shown in green), then the distances between foci were measured on autosomal bivalents that contain two or more MLH1 foci. (B, C) Examples of relative (B) and corresponding cumulative (C) frequency plots of gamma distributions. If there is no interference between MLH1 foci, an exponential frequency distribution is expected (gray lines). Deviation from exponential behavior indicates the existence of interference (red and blue lines): short and long distances become more rare and the spacing becomes more even (i.e., distances are tightly clustered). Curves were calculated using an average interfocus distance of 10 and the indicated increasing values for the shape parameter, ν. See text and [51] for further discussion.

Interference is strong in mice and other mammals: the majority of chromosome pairs form only a single crossover, and multiple crossovers on the same bivalent tend to be both widely and evenly spaced [51],[52]. Directly measuring crossover interference in mice requires analysis of viable progeny, precluding studies in sterile mutants. However, alternative methods have been developed for measuring cytological manifestations of interference, by measuring the distance between adjacent MLH1 foci [51] (Figure 7A). If MLH1 foci are randomly distributed relative to one another (i.e., if there is no interference), the distances between foci should show an exponential frequency distribution (gray curves in Figure 7B and 7C). Deviation from an exponential distribution provides a quantitative measure of the strength of interference [51] (red and blue curves in Figure 7B and 7C).

**Figure 8:**
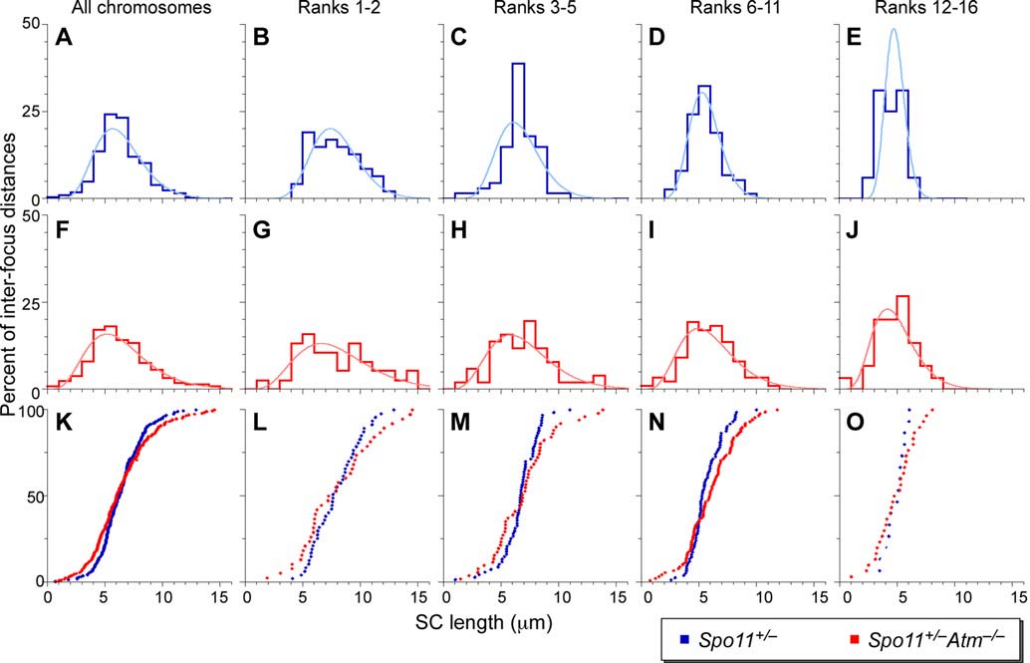
Decreased cytological interference on autosomes in *Spo11^+/−^Atm^−/−^* spermatocytes. Distances between pairs of adjacent MLH1 foci were measured on autosomes of pachytene spermatocytes. Panels A–E and F–J show the frequency distributions (step plots) of inter-focus distances for *Spo11^+/−^* (blue; 46 cells from 4 mice) and *Spo11^+/−^Atm^−/−^* (red; 38 cells from 4 mice), respectively. Best-fit gamma distributions are superimposed on each (smooth curves). Panels K–O show cumulative frequency plots to facilitate comparison of the two genotypes. The left column of graphs (A, F, K) pools data for all autosomes. The remaining columns show data for groups of similarly sized chromosomes, ranked from largest to smallest. Autosome size ranks 17–19 are excluded from this analysis because they rarely have more than a single MLH1 focus (see Table 1).

We examined MLH1 inter-focus distances in *Spo11^+/−^* and *Spo11^+/−^Atm^−/−^* pachytene spermatocytes (Figures 8 and S5). *Spo11^+/−^* spermatocytes showed the expected normal pattern of relatively even and wide spacing of MLH1 foci. Even spacing is revealed by the narrow distribution of inter-focus distances, with most pairs of MLH1 foci 4.5–9.5 μm apart (163 of 208 pairs, 78.4%). Note the tight clustering of values in frequency distribution plots (Figure 8A–8E) and the steeply sigmoidal shape of cumulative frequency plots of the same data (Figure 8K–8O). Wide spacing is revealed by the relative rarity of close foci: only 29 of 208 MLH1 focus pairs (13.9%) were ≤4.5 μm apart in *Spo11^+/−^* spermatocytes.

ATM-deficient cells showed a different pattern. The frequency distributions of inter-focus distances (Figure 8F–8J) appeared broader and flatter in *Spo11^+/−^Atm^−/−^* spermatocytes than in *Spo11^+/−^*, and cumulative frequency curves (Figure 8K–8O) were less steeply sigmoidal. Significantly fewer pairs of foci were in the range of 4.5–9.5 μm apart (135 of 212 pairs (63.7%); *p* = 0.001, Fisher's exact test). Thus, the spacing between foci is less regular in the absence of ATM. This difference is still seen if distances are normalized for total SC length (Figure S5 and data not shown), suggesting that the difference is not simply a consequence of the longer SCs resulting in more spread-out foci in ATM-deficient cells. Moreover, closely spaced foci occurred significantly more frequently in *Spo11^+/−^Atm^−/−^* spermatocytes, with 51 of 212 pairs separated by 4.5 μm or less (24.1%; *p* = 0.006, Fisher's exact test). Importantly, this result cannot be an artifact of longer SCs or the presence of gaps, because these would be expected to increase observed inter-focus distances, not decrease them. Indeed, the effect of ATM deficiency was even more pronounced if distances were measured as percent of SC length, which normalizes for the increase (Figure S5).

The same conclusions were drawn if we considered only chromosomes without obvious SC gaps (data not shown). Thus, differences between *Spo11^+/−^* and *Spo11^+/−^Atm^−/−^* interference patterns cannot be attributed to technical difficulties in accurately measuring distances caused by the presence of gaps. Moreover, the same conclusions were drawn if we considered inter-focus distances only on chromosomes with exactly two foci (data not shown). Thus, we cannot account for the increased occurrence of closely spaced foci in *Spo11^+/−^Atm^−/−^* spermatocytes as a trivial consequence of packing more foci into a limited space.

Frequencies of inter-focus distances can be approximately modeled by a gamma distribution, which provides an additional method to quantitatively assess cytological interference [(see [51] for detailed discussion). The gamma distribution that best fits the observed frequency distribution is characterized by a shape parameter (abbreviated “ν”), which can be regarded as a measure of the strength of interference between MLH1 foci: a value of ν = 1 implies no interference, whereas higher ν values indicate more regular spacing between foci, and thus stronger interference [51] (Figure 7B and 7C). Best-fit gamma distributions are shown as smooth curves in Figures 8A–8J and S5A–S5J, and ν values are given in Table 2. *Spo11^+/−^* spermatocytes showed high values of ν, comparable to previously described wild-type values [51]. In contrast, ATM-deficient cells showed an approximately 2-fold (or greater) reduction in ν values, indicating less regular spacing between MLH1 foci and thus less interference. The same conclusion is drawn whether considering all intervals together or each chromosome size group separately, and regardless of whether absolute distance (μm of SC) or normalized distance (percent of SC length) is used as the metric (Table 2). Moreover, the same conclusion is drawn if the ν values are corrected for the limited number of interfocus distances that can be observed on SCs of finite length (Table 2).

**Table 2:** Evidence for reduced cytological interference in *Spo11^+/−^Atm^−/−^* spermatocytes.

			μm of SC			% of SC Length		
Genotype	Chromosome size rank	N a	ν (SE) b	p c	ν (corr.) d	ν (SE) b	p c	ν (corr.) d
*Spo11^+/−^*	Total	206	9.3 (0.9)	0.25	6.3	17.9 (1.7)	0.00	12.2
	1–2	47	15.0 (3.1)	0.55	11.8	17.4 (3.5)	0.36	14.7
	3–5	67	12.3 (2.1)	0.015	10.2	22.9 (3.9)	0.00	20.5
	6–11	74	17.3 (2.8)	0.39	11.8	21.1 (3.4)	0.50	14.6
	12–16	15	34.7 (12.6)	0.27	18.2	47.2 (17.2)	0.11	25.1
*Spo11^+/−^Atm^−/−^*	Total	211	5.3 (0.5)	0.69	3.8	7.7 (0.7)	0.001	5.5
	1–2	38	5.9 (1.3)	0.73	4.9	8.5 (1.9)	0.22	7.2
	3–5	51	6.3 (1.2)	0.68	4.9	9.5 (1.8)	0.73	7.4
	6–11	88	5.9 (0.9)	0.33	4.4	6.3 (0.9)	0.15	4.6
	12–16	30	6.9 (1.7)	0.66	4.8	8.6 (2.2)	0.06	5.9

Pachytene spermatocyte spreads were immunostained for SYCP3 and MLH1. For autosomal bivalents with two or more MLH1 foci, distances between the foci were measured and expressed as absolute distance (μm of SC) or relative distance (% of SC length). Gamma distribution parameters that best fit the observed frequency distributions of inter-focus distances were calculated. Where indicated, bivalents were further subdivided into groups of similarly sized chromosomes as described in the text and the legend to Table 1.

a Number of interfocus distances analyzed.

b Shape parameter (ν) and standard error (SE). A larger value of ν indicates a more even spacing of MLH1 foci and thus greater cytological interference.

c Goodness-of-fit of the experimental data to the gamma distribution (high p value indicates better fit).

d Corrected shape parameter. The gamma distribution assumes theoretical limits of infinitely small and infinitely large interfocus distances, but the actual range of inter-focus distances that can be detected is limited by the resolution of light microscopy and by the finite length of each SC. Corrections for these limits were estimated as described in [51].

Taken together, these data reveal that cytological interference is reduced (but not absent) in *Spo11^+/−^Atm^−/−^* spermatocytes. These findings suggest that crossover interference is partially defective when ATM is not present.

## Discussion

In the absence of ATM, mouse spermatocytes and oocytes die by apoptosis during prophase of meiosis I, exhibiting profound defects in meiotic chromosome behavior [22],[24]. Remarkably, most of the spermatocyte defects are eliminated simply by halving *Spo11* gene dosage: instead of dying by apoptosis at pachynema (like *Atm^−/−^* and *Spo11^−/−^* single mutants), most *Spo11* ^+/−^ *Atm^−/−^* spermatocytes progress to metaphase I, and sometimes beyond [32]. Homologous synapsis, sex body formation, and crossing over are substantially, albeit incompletely, rescued. In this study, we took advantage of this intriguing phenotype to analyze the role of ATM in meiotic recombination. As discussed further below, our findings suggest previously undefined roles of ATM in crossover control and in promoting integrity of higher order chromosome structures.

How does *Spo11* heterozygosity rescue *Atm^−/−^* meiotic progression? Cytological and other evidence suggest that *Spo11^+/−^* spermatocytes form fewer DSBs than wild type (F. Cole, S. Keeney, and M. Jasin, unpublished observations). Given that ATM has an established role in meiotic DSB repair [20],[21],[32], a straightforward interpretation is that a reduced number of SPO11-generated DSBs is responsible for suppression of the meiotic DSB repair defects arising from ATM deficiency. Perhaps there is a threshold amount of DSBs below which another kinase (e.g., ATR) can partially substitute for ATM; above this threshold, the number of DSBs may exceed the capacity for this kinase to substitute. It is also possible that DSBs are formed in wild-type numbers in *Spo11^+/−^* mice but are delayed such that induction of ATR later in prophase is able to substitute for ATM [32]. Current findings do not allow us to distinguish between these and other possibilities.

*Atm^−/−^* spermatocytes, similar to many other mutants including *Spo11^−/−^*, *Dmc1^−/−^* and *Msh5^−/−^*, show pronounced defects in forming a bona fide sex body and transcriptionally silencing the X and Y chromosomes [20],[37]. Studies of these and other mouse mutants defective for MSCI strongly support the hypothesis that failure to silence the sex chromosomes is sufficient to trigger apoptosis of pachytene spermatocytes in stage IV tubules (reviewed in [37],[38]). Thus, the substantial restoration of sex body formation and MSCI in *Spo11^+/−^Atm^−/−^* spermatocytes may account for the suppression of *Atm^−/−^* pachytene apoptosis.

Although *Spo11^+/−^Atm^−/−^* spermatocytes progress further than *Atm^−/−^* single mutants, they are substantially eliminated at or prior to the first meiotic division. It is possible that apoptosis is triggered by a spindle checkpoint responding to the lagging chromosomes observed at metaphase I, which are likely to be the frequently achiasmate X and Y. Indeed, metaphase I spermatocyte apoptosis has been observed in several instances where one or a few achiasmate chromosomes are present because of chromosomal abnormalities [53]–[55], as well as in *Mlh1^−/−^* mice in which most chromosomes lack chiasmata [56]. Alternatively, or in addition, metaphase I apoptosis of *Spo11^+/−^Atm^−/−^* spermatocytes may be a response to unrepaired DSBs, whose presence is indicated by persistent γH2AX, RAD51, and RPA (data not shown).

The rescue of meiotic progression in *Spo11* ^+/−^ *Atm^−/−^* females was much less pronounced than in males. We have shown that ovarian follicle formation is particularly sensitive to the presence of unrepaired DNA damage [21]. Thus, even if meiotic prophase events were rescued to the same extent as in spermatocytes, it is possible that persistent DNA damage would preclude rescue of oocytes at this stage.

### ATM and Control of the Normal Number and Distribution of Crossovers

#### Forming an Obligate Crossover

In most organisms, nonexchange chromosomes occur rarely, giving rise to the concept of the obligate crossover [57] (see Introduction). The mammalian XY pair shares homology only within the very small PAR, and as a result, the crossover frequency in males is elevated >100-fold in the PAR over the genome average. DSB frequency in the PAR must be elevated at least 10-fold over the genome average to ensure that the PAR receives at least one DSB. (Up to 300 DSBs are estimated per 3×10^9^ bp per haploid genome, or 1 DSB per 10^7^ bp, while the mouse PAR is estimated to be <10^6^ bp long [4].) But it is also likely that a DSB(s) within this limited physical distance has a greater probability of being converted to a crossover than the “average” DSB on an autosome. Thus, we infer that one or more aspects of crossover control play a particularly important role within the PAR to ensure formation of the obligate crossover. By this reasoning, XY recombination should be uniquely sensitive to perturbations in crossover control as well as to other defects in interhomolog recombination more generally. Here, we demonstrate that *Spo11^+/−^Atm^−/−^* spermatocytes frequently contain an achiasmate X and Y pair. *Spo11^+/−^* spermatocytes have no such difficulty, hence this defect can be attributed specifically to the lack of ATM. While it is formally possible that the achiasmate XY configuration reflects a defect in sister chromatid cohesion, we favor the interpretation that ATM-deficient cells have difficulty in forming the obligate XY crossover because a crossover defect could account for frequent failure of XY homologous synapsis, as SC formation is thought to initiate at crossover-designated recombination sites (reviewed in [36],[49]).

Several factors contribute to forming an obligate crossover. At least one DSB must form per chromosome pair and the proper recombination partner (the homolog rather than the sister) must be located and engaged. Furthermore, differentiation of individual recombination events into crossovers must also be controlled. This control involves a “decision” early in the recombination reaction that determines whether a given DSB will become a crossover rather than a noncrossover, plus enforcement of this decision to ensure formation of the correct recombinant product [8],[11]. In principle, ATM could contribute to one or more of these processes. However, we consider it unlikely that a DSB deficit explains the frequent crossover failure in *Spo11^+/−^Atm^−/−^* spermatocytes: *Spo11* heterozygosity itself does not cause an XY crossover problem, and absence of ATM does not significantly decrease numbers of RAD51 foci in a *Spo11^+/−^* background, suggesting that ATM deficiency does not reduce DSB frequency. Therefore, it is possible that the XY defect reflects a requirement for ATM in promoting the XY crossover per se. There are several nonexclusive possibilities. ATM may be required for proper designation of a crossover outcome, either as part of the regulation of the crossover vs. noncrossover decision or as part of the response to that decision once it has been made. This model would implicate ATM in crossover control, consistent with effects on autosomes (see below) and consistent with the observation that orthologs of ATM/ATR promote crossover interference in budding yeast via phosphorylation of the single-stranded binding protein RPA [58]. Another possibility is that crossover designation occurs properly in the absence of ATM, but that there is a defect in later steps of recombination such that crossover or chiasma formation fails. Notably, however, there was no detectable increase in achiasmate autosomes, suggesting that ATM is not strictly required for maturing chiasmata.

A third possibility is that ATM is required for efficient homologous pairing and/or choice of partner for recombination (i.e., homolog versus sister). This hypothesis would place ATM function at or prior to the step when the homologous chromosome is located and engaged, i.e., before a decision is made about a crossover vs. noncrossover outcome. Further studies of the kinetics and efficiency of homologous pairing in normal and ATM-deficient mouse meiosis are necessary to distinguish between these possibilities. However, it is interesting to note that budding yeast ATM/ATR orthologs are required to suppress recombination between ectopic repeated sequences and to promote the normal bias toward interhomolog rather than intersister recombination [27],[28].

Why is there an obligate crossover defect on the XY but not on autosomes? One reasonable explanation is that the small size of the PAR makes this region uniquely sensitive to defects in any or all of the processes listed above. At ∼55–58 Mbp, even the smallest autosome (Chr19) is some 80 times as long as the PAR and forms on average 4–5 RPA and MSH4 foci, of which typically only one will give rise to a crossover [51]. The formation of multiple DSBs along each autosomal bivalent means that there are multiple opportunities to promote pairing and to successfully execute chiasma formation, such that a partial defect in pairing, in formation of crossovers, or in regulation of crossover number and distribution might have little effect on the frequency of non-exchange bivalents. In contrast, the XY pair may have as little as a single DSB within the PAR in any one cell, which would place a much greater premium on efficient execution of all of the steps that lead to chiasma formation.

#### Crossover Interference

Meiotic recombination is also subject to one or more activities that depress the total number of crossovers and ensure that crossovers are widely and evenly spaced. *Spo11^+/−^Atm^−/−^* spermatocytes had a small but significant defect in cytological interference, suggesting that ATM is required for normal crossover interference. We cannot exclude the possibility that the increased heterogeneity in inter-MLH1 focus distances is simply a consequence of altered axis structure instead of altered crossover interference (e.g., if ATM deficiency causes the SC to appear stretched in some places and compressed in others, such that distances along the axis do not share the same relationship with DNA distances as in wild type). However, such a scenario would not explain the accompanying increase in the number of autosomal MLH1 foci in *Spo11^+/−^Atm^−/−^* spermatocytes. We therefore favor the interpretation that ATM is partially required for both of these suppressive aspects of crossover control. One possibility is that ATM-deficient cells form a small number of “extra” autosomal crossovers that do not display the normal constraints on number and position. Such an outcome could occur if ATM is involved in making or enforcing the crossover vs. noncrossover decision locally at recombination sites, and/or if it is involved in propagation or response to crossover-inhibiting signals between adjacent recombination sites.

Lack of ATM causes defects in both crossover-promoting (the obligate XY crossover) and crossover-suppressing (interference) aspects of meiotic recombination. This feature of the results is consistent with the hypothesis that both positive and negative aspects of crossover control are closely related processes [3],[5],[7],[49].

### Connecting Crossover Control and Chromosome Structures

*Spo11^+/−^Atm^−/−^* spermatocytes exhibited numerous defects in chromosome axes. It is possible that the structural flaws reflect defects in axis morphogenesis, but as discussed below there is also reason to consider that the lack of ATM causes defects in axis stability. Previous studies noted chromosome fragmentation in *Atm^−/−^* spermatocytes but were unable to distinguish whether this defect was an indirect effect of arrest and apoptosis in early to mid prophase [22]. Since progression through meiotic prophase I is substantially rescued in *Spo11^+/−^Atm^−/−^* spermatocytes, our results indicate that axis defects are more directly tied to the lack of ATM.

The crossover and chromosome axis defects in *Spo11^+/−^Atm^−/−^* spermatocytes may be separate. However, considerations about the relationship between meiotic recombination and higher order chromosome structures lead us to speculate instead that these defects may be manifestations of a single underlying problem. In many organisms, mutations affecting chromosome structure proteins perturb meiotic recombination and, conversely, mutations affecting recombination factors perturb chromosome structures [reviewed in 36,49]. Moreover, cytological and molecular studies reveal that meiotic recombination occurs in close spatial coordination with chromosome axes (reviewed in [49]). Taken together, these observations reveal functional connections between recombination and axes. It has been argued that these connections are important for establishing a functional chiasma, because a chiasma is more than just a crossover at the DNA level—a chiasma also involves higher order chromosome structure changes, including exchange of the chromosome axes and local separation of sister chromatids [5],[49],[59].

In order for chromosome structures and recombination events to develop in parallel, signals coordinating these processes must be transduced in both directions between the axes and the recombination machinery. Moreover, chromosome axes are likely to participate directly in crossover control by providing a conduit for an interference signal that governs distribution of crossovers [5],[60]. We propose that ATM kinase activity generates or transduces one or more of these signals. Consistent with this interpretation, mutations of *Mre11* and *Nbs1* that attenuate ATM signaling also cause crossover control defects in mouse spermatocytes [46]. Relevant phosphorylation targets remain to be identified, but might include histones, structural components of the axes, and/or recombination proteins (see also [27],[58]). Non-catalytic (i.e., kinase-independent) functions of ATM are also possible [61].

This model suggests how axis and recombination perturbations could both arise from absence of ATM. Sites of ongoing recombination are also places where axes are locally destabilized, for example showing buckling or twisting of the axes (reviewed in [49],[62]). If *Atm^−/−^* mutants are defective for interactions between recombinosomes and the axes (e.g., if ATR is only partly effective as a substitute), then correlated defects would be expected in all of the processes that depend on these interactions. If correct, this model predicts that axial interruptions in *Spo11^+/−^Atm^−/−^* spermatocytes occur specifically at sites where DSBs have occurred. The observed correlation between chromosomal anomalies and persistent γH2AX, RAD51, and RPA foci at pachynema in these mice is consistent with this prediction. Moreover, we found that axis defects that result in overt chromosome fragmentation in the absence of ATM are spatially correlated with chromosomal regions where crossover control is known to play an important role—the short SC fragments in *Spo11^+/−^Atm^−/−^* spermatocytes were usually derived from the distal tips of chromosomes, and there is a known preference in spermatocytes for one (or the only) crossover on a bivalent to be located distally [12],[51]. This nonrandom positioning is thought to be another manifestation of crossover control [51],[63]. Thus, the position of fragmentation is consistent with our hypothesis that axis and crossover control defects are functionally connected.

The meiotic cell's ability to coordinate multiple molecular processes spanning size scales that differ by orders of magnitude is truly remarkable. The unexpected rescue by *Spo11* hemizygosity of meiotic prophase progression in *Atm^−/−^* spermatocytes has allowed us to identify ATM as a prime candidate to be directly involved in this unique feature of meiotic chromosome dynamics.

## Materials and Methods

### Mice

*Spo11^−/−^* and *Atm^−/−^* mice were as previously described [16],[22] on a C57Bl/6×129/Sv mixed background. To minimize variability from strain background, experimental animals were compared to controls from the same litter or from the same matings involving closely related parents. Each analysis was done with 2–4 *Spo11^+/−^Atm^−/−^* experimental animals (except for TOPBP1 staining, Figure S2). In each case, experimental animals were matched with 2–4 *Spo11^+/−^* controls, except for RAD51 focus counts (text); phospho-Pol II staining (Figure 4A), STAG3 staining (Figure 5D), evaluation of chromosome continuity by combined FISH/immunofluorescence (Figure 5E), and TOPBP1 staining (Figure S2). Importantly, all of the patterns described above for XY synapsis/chiasma defects, autosomal MLH1 numbers and distributions, and chromosome axis defects were reproducibly observed in multiple sib-pair comparisons. No significant variations were observed in between-individual or between-litter comparisons of animals with the same *Spo11/Atm* genotype. Genotyping was performed by PCR of tail tip DNA as previously described [21]. Experiments conformed to relevant regulatory standards and were approved by the MSKCC Institutional Animal Care and Use Committee.

### Cytology, Histology, and FISH

Testis cell preparations were prepared for surface spreading and sectioning as described [20] from 2–4 month-old mice unless otherwise stated. Indirect immunofluorescence analysis of spread chromosomes was performed using described methods and antibodies [20]. Additional primary antibodies were rabbit anti-MLH1 (Calbiochem PC56T), 1∶75 dilution; rabbit anti-RAD51 (Oncogene), 1∶250; rabbit anti-TRF1 (generously provided by T. de Lange, Rockefeller Univ.), 1∶200; guinea pig anti-STAG3 (generous gift of C. Höög, Karolinska Institute), 1∶30; and CREST serum to detect centromeres (generous gift of P. Moens, York University), 1∶500. For RAD51 and MLH1 focus counts, nuclei were staged according to the extent of SYCP3 staining and synapsis. Leptonema was defined as having short, unsynapsed SYCP3 fragments. Early zygonema was defined as <50% synapsis and late zygonema was defined as >50% but less than 100% synapsis. Only nuclei with at least 19 autosomal MLH1 foci were considered for MLH1 counts, and only RAD51 and MLH1 foci that co-localized with SYCP3 staining were counted.

Detailed methods for testis sectioning and immunohistochemistry are described elsewhere [20]. Briefly, for histological analysis, sections were stained by periodic acid-Schiff (PAS) and hematoxylin. Spermatogenic staging of PAS-stained seminiferous tubule sections was as described [33]. For immunohistochemistry, anti-phospho-histone H3 antibody (Upstate Cell Signaling) was used at 5 μg/ml and detected with HRP-conjugated secondary antibodies using DAB as a substrate; slides were counter-stained with hematoxylin.

For analysis of meiotic spindles, 30-μm testis sections were placed on poly-L-lysine (Sigma) coated slides, and dried 2 hr, followed by a 2 hr incubation at 37°C. Slides were post-fixed 10 min in cold methanol, washed twice with PBS, then blocked for 20 min with antibody dilution buffer [20], and washed three times with PBS before incubation overnight at 4°C with anti-β-tubulin antibody (Sigma T4026) at 1∶200 dilution. Coverslips were mounted using ProLong Gold (Molecular Probes) containing DAPI. Images were analyzed using a confocal imaging system (Zeiss).

Combined immunofluorescence/FISH was performed as described [20] using FITC-conjugated X chromosome paint (Cambio, UK) and coumarin-conjugated (ENZO) Y-specific repetitive BAC probe Ct7-590p11 (Invitrogen). SpectrumGreen-conjugated (Vysis, Abbott Labs) PAR-specific probe was prepared from mouse BAC RP24-500I4 (CHORI). Paints for chromosomes 10 and 3 were from Cambio. SKY was performed as described [64]. Metaphase cells were documented with a Nikon E800, and images were analyzed using the SKYview 2.1.1 software.

### Interference and Statistical Analyses

Autosomal SC lengths and MLH1 focus positions were recorded using MicroMeasure, version 3.01 (http://www.colostate.edu/Depts/Biology/MicroMeasure). MLH1 position was measured from the centromeric end of the chromosome as revealed by the brighter DAPI staining of pericentromeric heterochromatin. Once SC length and the position of each MLH1 focus were obtained, the SCs in each spread were rank-ordered based on their absolute length, from rank 1 (longest) to rank 19 (shortest). Similarly ranked SCs were grouped to allow comparison of similar chromosomes between cells and genotypes. Based in part on published analyses of autosomal SC sizes using combined FISH and immunofluorescence [52], we chose the following groupings: ranks 1–2 (i.e., the two longest SCs in each spread), ranks 3–5, ranks 6–11, ranks 12–16, and ranks 17–19. For SCs containing gaps, the length of the gap was subtracted from the total length of the bivalent. Whenever possible for SC fragments, the lengths of fragments that appeared to originate from the same bivalent were combined to estimate the complete SC length for ranking purposes. Best fits of frequency distributions of MLH1 inter-focus distances to the gamma distribution were calculated using the GenStat software package (VSN International Ltd, Hemel Hempstead, UK), as described [51]. Correction was applied as described [51] to adjust ν values for the limited number of interfocus distances that can be measured (see Table 2). Other statistical tests were as specified in the text. We applied the non-parametric Mann-Whitney U Test to total numbers of MLH1 foci per cell to avoid the need to assume that the data were normally distributed. However, similar conclusions as to statistical significance were drawn if a t test was used instead (data not shown). Two-by-two contingency tables were subjected to two-tailed Fisher's exact tests. For larger contingency tables, a log-likelihood test for heterogeneity (G test) was applied.

## Supporting Information

Figure S1 Additional examples of seminiferous tubule sections from *Spo11^+/−^Atm^−/−^* mice. (A) Morphologically normal pachytene spermatocytes (p) are seen at stage IV and later stages. (B) Round spermatids (rs) are also observed, as are abnormal spermatids which may be diploid (ds) (inset). (C) Apoptosis of metaphase cells in stage XII tubules was frequent in S*Spo11^+/−^Atm^−/−^* testis, as revealed by TUNEL assay (brown stain).
(3.37 MB TIF)

Figure S2 Sex body formation does not require ATM or XY synapsis. (A–D) Pachytene chromosome spreads of *Spo11^+/−^Atm^−/−^* spermatocytes (n = 51) were analyzed by immunofluorescence for SYCP3 in conjunction with FISH for the XY pair and γH2AX immunofluorescence. In all cases where the sex chromosomes are adjacent to one another, they are included within a common γH2AX domain whether they are synapsed (not shown) or unsynapsed (A,B). When the sex chromosomes are widely separated, two γH2AX signals (arrowheads) are generally observed (7/8 spermatocytes) (C,D). (E, F) Pachytene chromosome spreads of *Spo11^+/−^Atm^−/−^* spermatocytes were analyzed by immunofluorescence for additional sex body components. Even when the XY pair is not synapsed, they are included within a common NBS1 (E) or TOPBP1 (F) domain.
(2.06 MB TIF)

Figure S3 γH2AX staining at leptonema and zygonema is not rescued in *Spo11^+/−^Atm^−/−^* spermatocytes, while at diplonema puffs of γH2AX are observed. Chromosome spreads from wild type and *Spo11^+/−^Atm^−/−^* testis were stained for SYCP3 and γH2AX. Leptotene spermatocytes from *Spo11^+/−^Atm^−/−^* mice show little or no γH2AX staining (compare A and B), while zygotene spermatocytes have reduced levels of γH2AX relative to wild type (compare C and D), similar to *Atm^−/−^* spermatocytes [20]. Diplotene *Spo11^+/−^Atm^−/−^* spermatocytes have persistent puffs of γH2AX on some autosomes (compare E and F).
(2.09 MB TIF)

Figure S4 SCs are longer on average in *Spo11^+/−^Atm^−/−^* spermatocytes, but most cells show SC lengths within the range found in normal cells. SC lengths for autosomal bivalents in pachytene cells were summed to obtain a total SC length per cell. Bars show means±sd.
(0.03 MB TIF)

Figure S5 Decreased cytological interference on autosomes in *Spo11^+/−^Atm^−/−^* spermatocytes. Distances between pairs of MLH1 foci are plotted as in Figure 8, but normalized to SC length. Panels A–E and F–J show the frequency distributions (step plots) of inter-focus distances for *Spo11^+/−^* (blue) and *Spo11^+/−^Atm^−/−^* (red), respectively. Best-fit gamma distributions are superimposed on each (smooth curves). Panels K–O show cumulative frequency plots to facilitate comparison of the two genotypes. The left column of graphs (A, F, K) pools data for all autosomes. The remaining columns show data for groups of similarly-sized chromosomes, ranked from largest to smallest. Autosome size ranks 17–19 are excluded from this analysis because they rarely have more than a single MLH1 focus (see Table 1).
(0.11 MB TIF)

Table S1 Autosomal SCs are longer on average in ATM-defective spermatocytes.
(0.04 MB DOC)
