# Supplementary figures and images for: ATM Promotes the Obligate XY Crossover and both Crossover Control and Chromosome Axis Integrity on Autosomes
Source: PLoS Genet. 2008 May 23;4(5):e1000076. doi: 10.1371/journal.pgen.1000076 (PMC2374915; doi:10.1371/journal.pgen.1000076)

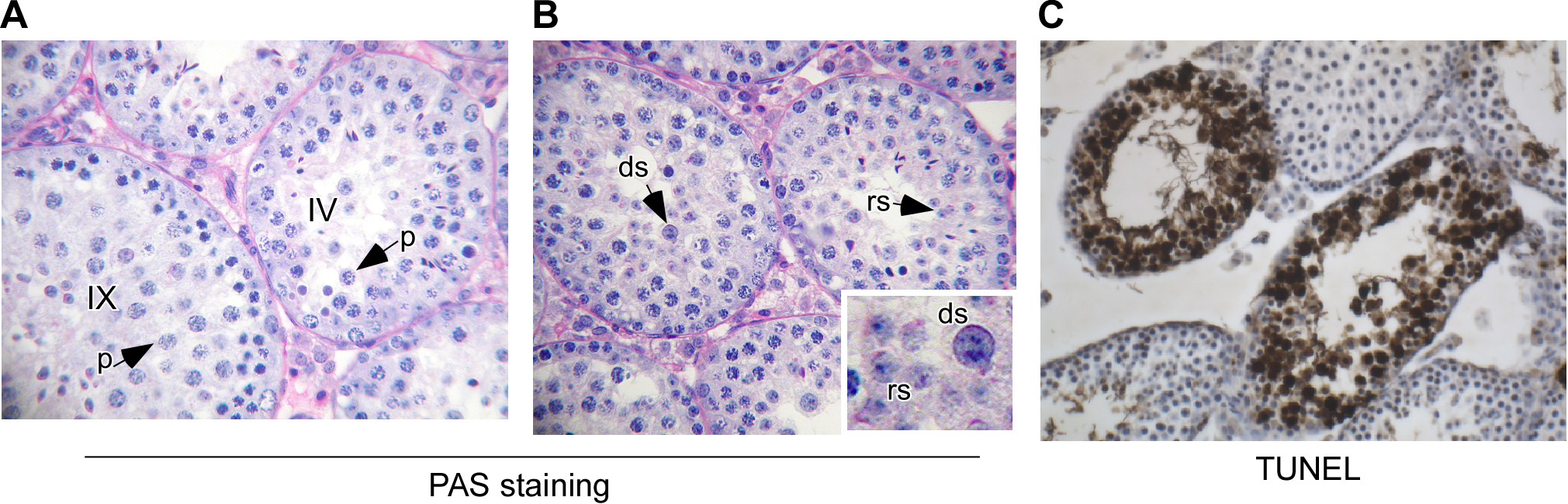

Supplement: Figure S1 — Additional examples of seminiferous tubule sections from Spo11+/−Atm−/− mice. (A) Morphologically normal pachytene spermatocytes (p) are seen at stage IV and later stages. (B) Round spermatids (rs) are also observed, as are abnormal spermatids which may be diploid (ds) (inset). (C) Apoptosis of metaphase cells in stage XII tubules was frequent in SSpo11+/−Atm−/− testis, as revealed by TUNEL assay (brown stain). (3.37 MB TIF) [file pgen.1000076.s001.tif]

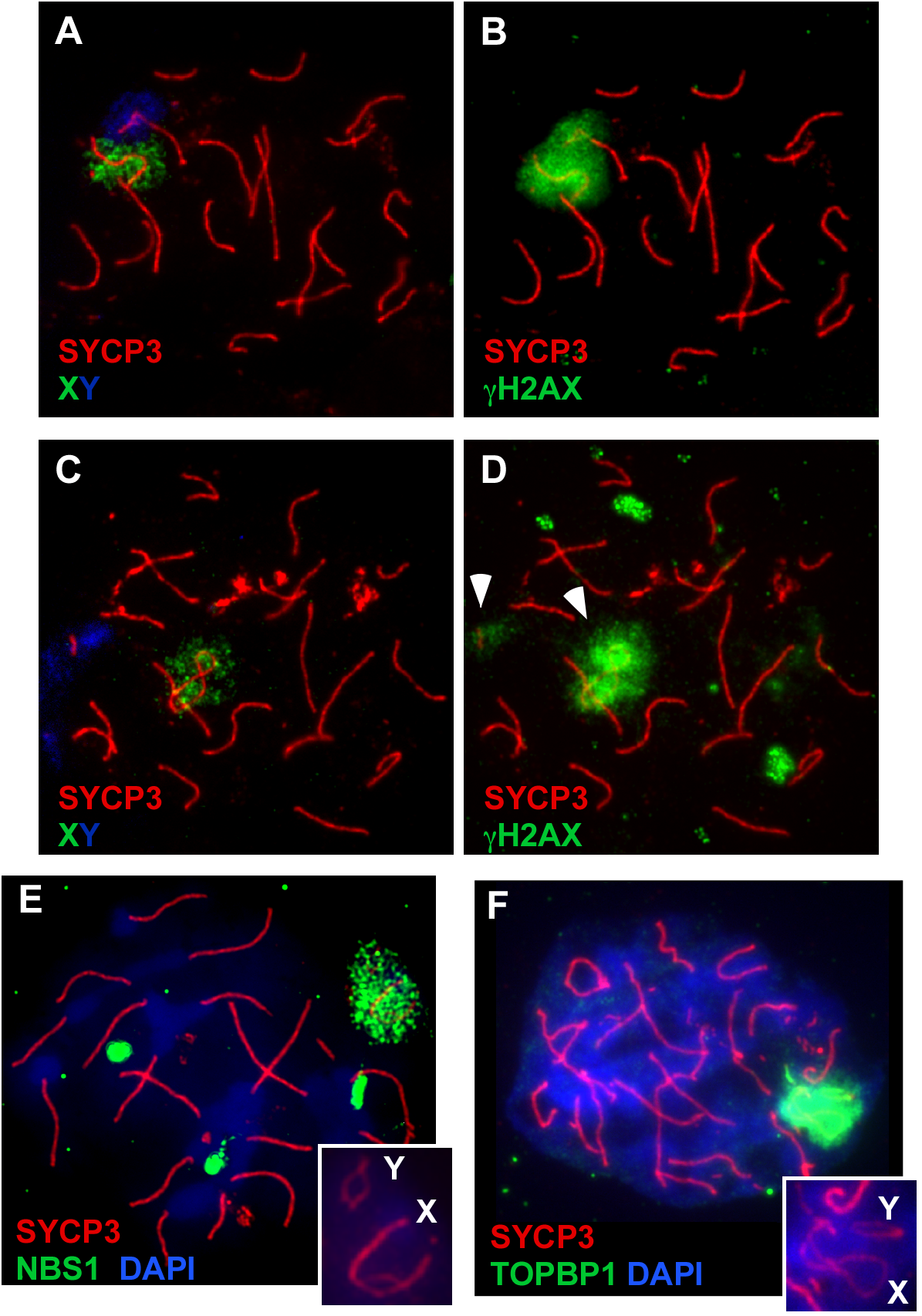

Supplement: Figure S2 — Sex body formation does not require ATM or XY synapsis. (A–D) Pachytene chromosome spreads of Spo11+/−Atm−/− spermatocytes (n = 51) were analyzed by immunofluorescence for SYCP3 in conjunction with FISH for the XY pair and γH2AX immunofluorescence. In all cases where the sex chromosomes are adjacent to one another, they are included within a common γH2AX domain whether they are synapsed (not shown) or unsynapsed (A,B). When the sex chromosomes are widely separated, two γH2AX signals (arrowheads) are generally observed (7/8 spermatocytes) (C,D). (E, F) Pachytene chromosome spreads of Spo11+/−Atm−/− spermatocytes were analyzed by immunofluorescence for additional sex body components. Even when the XY pair is not synapsed, they are included within a common NBS1 (E) or TOPBP1 (F) domain. (2.06 MB TIF) [file pgen.1000076.s002.tif]

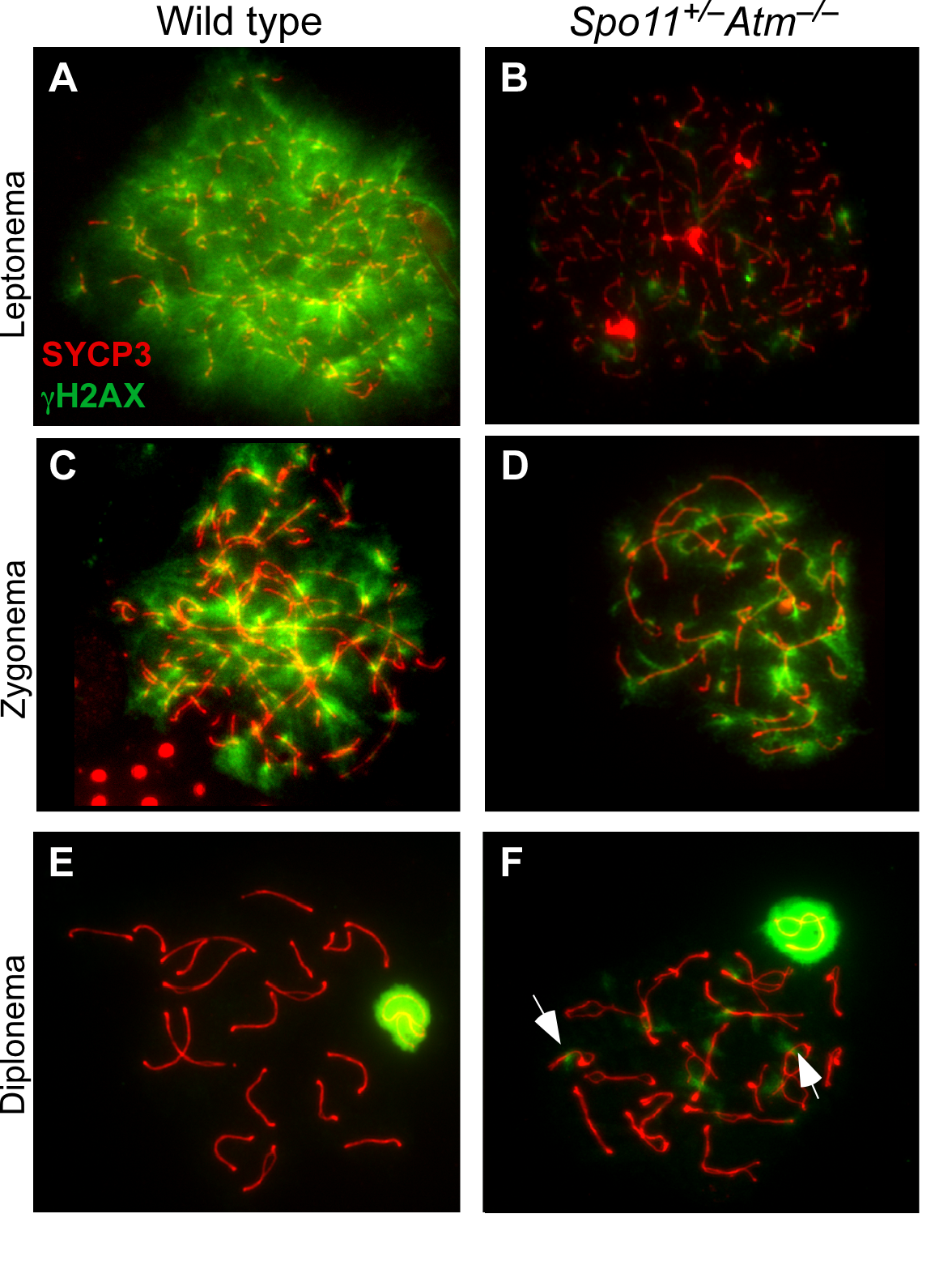

Supplement: Figure S3 — γH2AX staining at leptonema and zygonema is not rescued in Spo11+/−Atm−/− spermatocytes, while at diplonema puffs of γH2AX are observed. Chromosome spreads from wild type and Spo11+/−Atm−/− testis were stained for SYCP3 and γH2AX. Leptotene spermatocytes from Spo11+/−Atm−/− mice show little or no γH2AX staining (compare A and B), while zygotene spermatocytes have reduced levels of γH2AX relative to wild type (compare C and D), similar to Atm−/− spermatocytes [20]. Diplotene Spo11+/−Atm−/− spermatocytes have persistent puffs of γH2AX on some autosomes (compare E and F). (2.09 MB TIF) [file pgen.1000076.s003.tif]

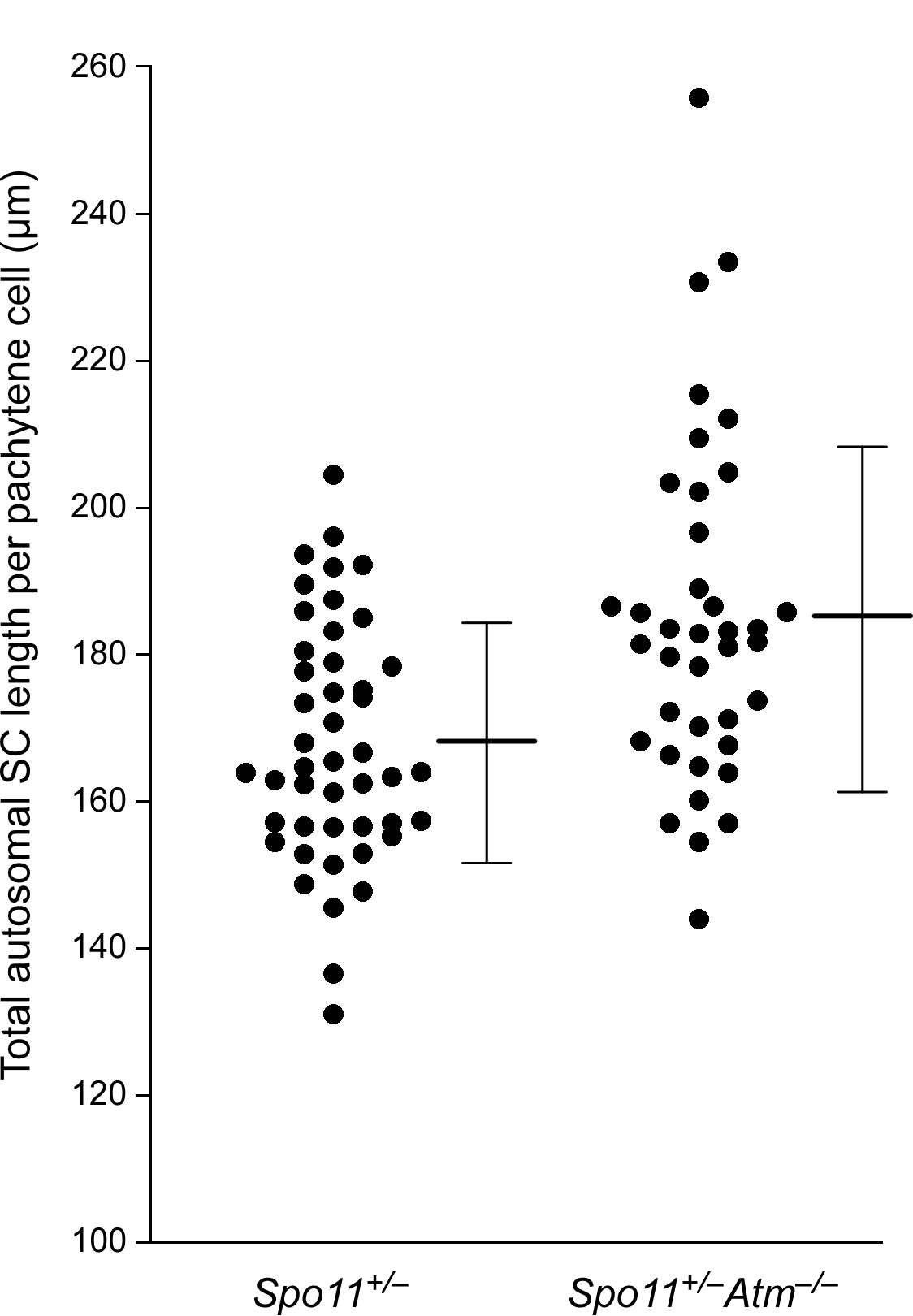

Supplement: Figure S4 — SCs are longer on average in Spo11+/−Atm−/− spermatocytes, but most cells show SC lengths within the range found in normal cells. SC lengths for autosomal bivalents in pachytene cells were summed to obtain a total SC length per cell. Bars show means±sd. (0.03 MB TIF) [file pgen.1000076.s004.tif]

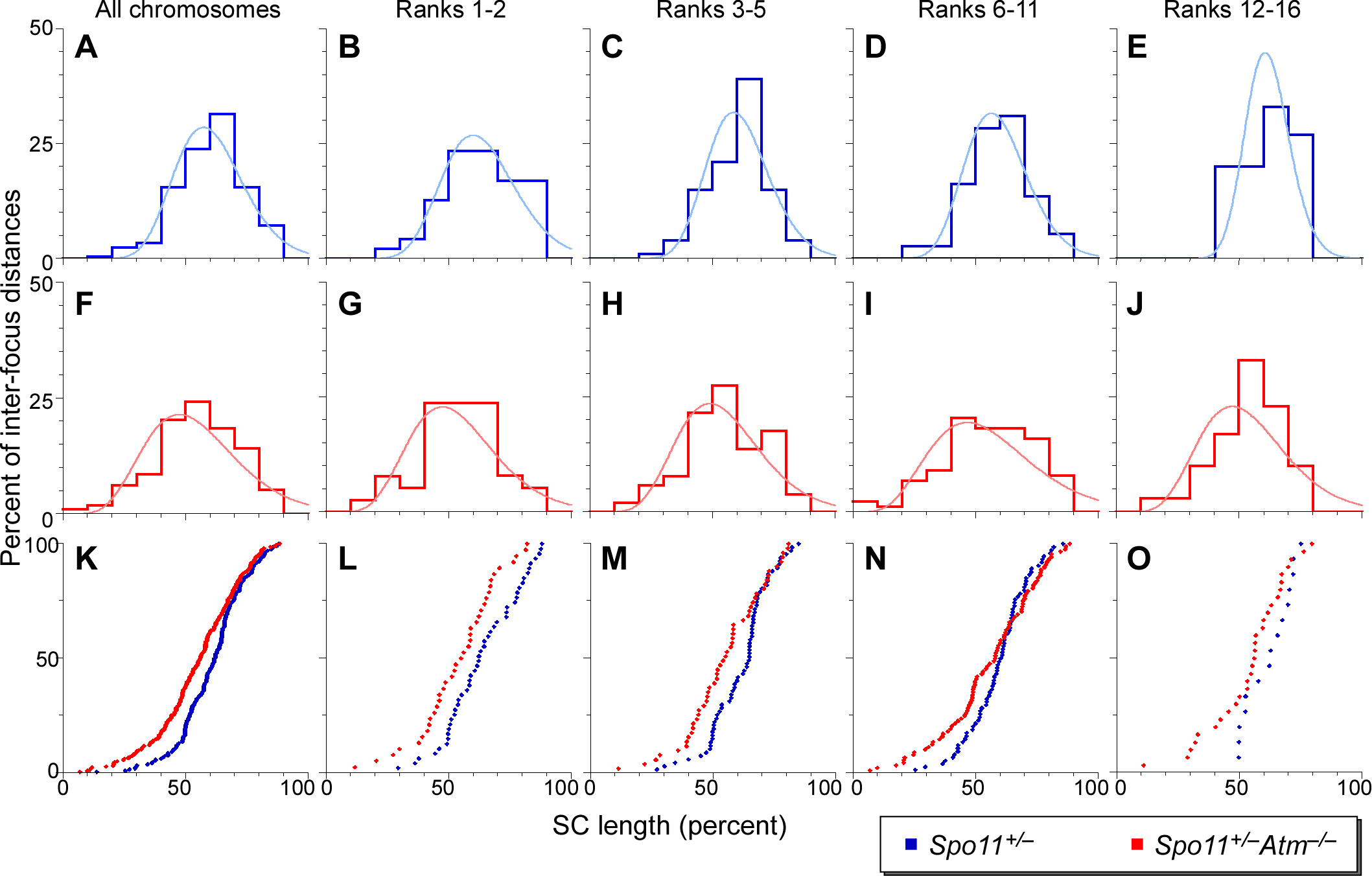

Supplement: Figure S5 — Decreased cytological interference on autosomes in Spo11+/−Atm−/− spermatocytes. Distances between pairs of MLH1 foci are plotted as in Figure 8, but normalized to SC length. Panels A–E and F–J show the frequency distributions (step plots) of inter-focus distances for Spo11+/− (blue) and Spo11+/−Atm−/− (red), respectively. Best-fit gamma distributions are superimposed on each (smooth curves). Panels K–O show cumulative frequency plots to facilitate comparison of the two genotypes. The left column of graphs (A, F, K) pools data for all autosomes. The remaining columns show data for groups of similarly-sized chromosomes, ranked from largest to smallest. Autosome size ranks 17–19 are excluded from this analysis because they rarely have more than a single MLH1 focus (see Table 1). (0.11 MB TIF) [file pgen.1000076.s005.tif]
