# Supplementary material for: ATM Promotes the Obligate XY Crossover and both Crossover Control and Chromosome Axis Integrity on Autosomes
Source: PLoS Genet. 2008 May 23;4(5):e1000076. doi: 10.1371/journal.pgen.1000076 (PMC2374915; doi:10.1371/journal.pgen.1000076)
Supplement: Table S1 — Autosomal SCs are longer on average in ATM-defective spermatocytes. (0.04 MB DOC) [file pgen.1000076.s006.doc]

**Table S1. Autosomal SCs are longer on average in ATM-defective spermatocytes.**

|  | *Spo11^+/–^* | | | *Spo11^+/–^Atm^–/–^* | | |
| --- | --- | --- | --- | --- | --- | --- |
| Chromosome size ranks | SC length (μm) ^a^ | % total SC ^b^ | N ^c^ | SC length (μm) ^a^ | % total SC ^b^ | N ^c^ |
| 1–2 | 12.7 ± 1.8 | 7.6 | 66 | 13.6 ± 2.8 | 7.4 | 54 |
| 3–5 | 10.9 ± 1.5 | 6.5 | 99 | 11.7 ± 2.1 | 6.3 | 81 |
| 6–11 | 9.3 ± 1.2 | 5.5 | 198 | 10.0 ± 1.7 | 5.4 | 162 |
| 12–16 | 7.6 ± 1.0 | 4.5 | 165 | 8.2 ± 1.4 | 4.4 | 135 |
| 17–19 | 5.6 ± 1.0 | 3.3 | 99 | 6.1 ± 1.3 | 3.3 | 81 |

Pachytene spermatocyte spreads were immunostained for SYCP3 and lengths of autosomal SCs were measured (46 cells from 4 mice (*Spo11^+/–^*) and 38 cells from 4 mice (*Spo11^+/–^Atm^–/–^*). The bivalents in each spread were rank-ordered by length from 1 (largest) to 19 (smallest), then divided into groups of similarly sized chromosomes. Note that each of the chromosomes in each size class makes up essentially the same percentage of total SC length in both genotypes, indicating that the increase in average total SC length in *Spo11^+/–^Atm^–/–^* spermatocytes is uniform across all autosomes.

a, mean ± standard deviation for each chromosome in the indicated size rank.

b, the mean SC length divided by the average total SC length per cell (see text), multiplied by 100.

c, total number of chromosomes analyzed for the indicated size rank.
